# Complete analytic solutions for convection-diffusion-reaction-source equations without using an inverse Laplace transform

**DOI:** 10.1038/s41598-020-63982-w

**Published:** 2020-05-15

**Authors:** Albert S. Kim

**Affiliations:** Civil and Environmental Engineering, University of Hawaii at Manoa, 2540 Dole Street Holmes 383, Honolulu, Hawai’i 96822 USA

**Keywords:** Chemical engineering, Applied physics

## Abstract

Transient mass-transfer phenomena occurring in natural and engineered systems consist of convection, diffusion, and reaction processes. The coupled phenomena can be described by using the unsteady convection-diffusion-reaction (CDR) equation, which is classified in mathematics as a linear, parabolic partial-differential equation. The availability of analytic solutions is limited to simple cases, e.g., unsteady diffusion and steady convective diffusion. The CDR equation has been considered analytically intractable, depending on the initial and boundary conditions. If spatial adsorption and desorption of matter are super-positioned in the CDR equation as sink and source functions, respectively, then the governing equation becomes an unsteady convection-diffusion-reaction-source (CDRS) equation, of which general solutions are unknown. In this study, a general 1D analytic solution of the CDRS equation is obtained by using a one-sided Laplace transform, by assuming constant diffusivity, velocity, and reactivity. This paper also provides a general formalism to derive 1D analytic solutions for Dirichlet/Dirichlet and Dirichlet/Neumann boundary conditions. Derivations of the analytic solutions are found to be straightforward if a combination of the source function and the initial concentration provide a non-zero singularity pole of inverse Laplace transform.

## Introduction

In models of transport phenomena, the mass-transfer rate at a given location is ascribed to diffusion, convection (or advection), reaction, and sources/sinks, which are ubiquitous in a plethora of natural and engineered processes. Diffusion can be described mathematically by using the transition probability describing locally hopping molecules^1^, random fluctuating forces satisfying the dissipation-fluctuation theorem^2,3^, and random walk phenomena caused by irreversible increases in entropy^4,5^. Convective transport originates from the motion of solute-carrying fluid, as obtained from the continuity equation. A chemical reaction is depicted as a continuous transformation of solutes toward a chemical equilibrium in a bulk phase. Finally, source and sink functions are ascribed to transporting masses that are either created or annihilated at specific locations in the environment. Obtaining rigorous solutions of the coupled phenomena is of great importance in various scientific and engineering disciplines. In past decades, analytic approaches to the coupled transport phenomena has been limited to coupled advection – diffusion without reactions: 1D unsteady in an open channel with spatially varying velocity and diffusivity^6^, 3D steady in a planetary layer (semi-analytic)^7^, and 1D unsteady with variable coefficients in semi – infinite media^8^.

A full mathematical expression of the above four mass transfer mechanisms is known as the convection-diffusion-reaction-source (CDRS) equation, wherein a sink can be expressed as a negative source. Specifically, a 1D-unsteady CDRS equation may be written as

1 $$\frac{\partial C}{\partial t}={D}_{0}\frac{{\partial }^{2}C}{\partial {x}^{2}}-{V}_{0}\frac{\partial C}{\partial x}-{K}_{0}C+S(x)$$

where $C(x,t)$ is the concentration at time $t$ and position $x$, and ${D}_{0}$ and ${V}_{0}$ are the constant diffusion coefficient and convective velocity, respectively, ${K}_{0}$ is a first-order reaction constant, and $S(x)$ is a source function. Because Eq. (1) is a parabolic partial differential equation, if Dirichlet boundary conditions (BCs) are assumed, a specific solution depends on an initial condition (IC) expressed as $C(x,t=0)={C}_{I}(x)$ and two BCs: one at the inlet, $C({x}_{0},t)={C}_{0}(t)$, and the other at the outlet, $C({x}_{1},t)={C}_{1}(t)$, where ${x}_{0} < {x}_{1}$ is given for convenience. For example, Dirichlet-type conditions of finite ${C}_{0}\ne 0$ and ${C}_{1}\mathrm{=0}$ indicate a non-zero concentration at the inlet and a perfect sink of the transferring mass at the outlet, respectively. In contrast, if a semi-infinite domain is considered such as ${x}_{1}\to \infty$, the Dirichlet BC of ${C}_{1}\mathrm{=0}$ is often switched to Neumann-type BC, i.e., ${[\partial C/\partial x]}_{x={x}_{1}}\to 0$, which is typically known as the zero-flux or exit BC. In the applied mathematics literature, a number of unsteady diffusion problems have been solved analytically using Green’s function and Laplace transform (LT) techniques^9^ with convection phenomena often discarded, especially, in long-term diffusion phenomena. For mathematical simplicity, Eq. (1) can be rewritten as

2 $$\frac{{\rm{\partial }}\phi }{{\rm{\partial }}\tau }=\frac{{{\rm{\partial }}}^{2}\phi }{{\rm{\partial }}{\xi }^{2}}-2\lambda \frac{{\rm{\partial }}\phi }{{\rm{\partial }}\xi }-\kappa \phi +\sigma (\xi )$$

by using dimensionless quantities defined as $\phi (\xi ,\tau )=C(x,t)/{C}_{\infty }$, $\tau =t{D}_{0}/{L}^{2}$, $\xi =x/L$, ${\rm{Pe}}=2\lambda =L{V}_{0}/{D}_{0}$, $\kappa ={K}_{0}{C}_{0}{L}^{2}/{D}_{0}$, and $\sigma =S{L}^{2}/{D}_{0}$, where ${C}_{\infty }$ is a reference (often bulk or initial) concentration, $L$ is the length scale of the spatial domain, Pe is the Peclet number, and the factor 2 of $\lambda$ is for mathematical convenience. In groundwater research, 1D sub-surface domains are either bounded $(0 < \xi  < 1)$ or semi-infinite $(0 < \xi  < +\,\infty )$. In this dimensionless analysis, the inverse time scale ${\tau }^{-1}$ and the dimensionless reactivity $\kappa$ quadratically increase with respect to $L$, i.e., $\tau \propto {L}^{-2}\,{\rm{and}}\,\kappa \propto {L}^{2}$ so that $\kappa \tau$ is independent of the domain length scale, $L$. In the limit of $L\to \infty$, an effect of diverging $\lambda$ in Eq. (2) is often nullified by the induced zero-flux condition at the outlet.

In this work, a general analytic solution of the CDRS Eq. (1) is obtained by using a one-sided LT without conducting the inverse LT explicitly. The steady-state solution, denoted ${\phi }_{{\rm{ss}}}(\xi )$, is obtained by using the residue theorem in complex analysis at the primary singular pole. The unsteady part of the full solution is obtained as a product of a spatial and a transient function, using the initial concentration, defined as ${\mu }_{I}(\xi )\equiv \phi (\xi ,\tau =0)$ and the source function $\sigma (\xi )$, both in dimensionless forms. If ${\mu }_{I}(\xi )$ and $\sigma (\xi )$ have forms of hyperbolic or sinusoidal functions, then the general analytic solution is obtained simply by identifying the secondary singularity pole of the LT ${\mathcal L} [\phi (\xi ,\tau )]$ in the complex domain. Several representative examples available in the literature were reproduced to confirm the analytical rigor and numerical accuracy of the current method. The current solution method, however, still contains a discontinuity due to the discrepancy between the IC and BC(s) at boundaries, which can be technically resolved by using a Fourier series.

## Convection-Diffusion Problems Revisited

Throughout the manuscript, ${\phi }_{0}$ and ${\phi }_{1}$ indicates constant dimensionless concentrations at the inlet $(\xi =0)$ and outlet $(\xi =1)$, and ${\mu }_{0}$ denotes especially the constant initial concentration (without $\xi$ dependence).

### Solution for Dirichlet Boundary Conditions

**Figure 1:**
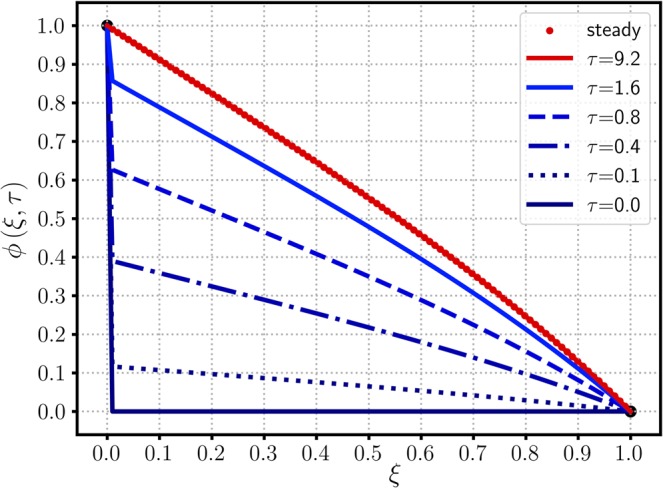
The time-evolution of $\phi (\xi ,\tau )$ for various $\tau$ values versus $\xi$. Parameters include ${\phi }_{0}\mathrm{=1}$, ${\phi }_{1}\mathrm{=0}$, ${\mu }_{0}\mathrm{=0}$, $\lambda \,\mathrm{=\; 1/2}({\rm{Pe}}\,\mathrm{=\; 1})$ and $\kappa \,\mathrm{=\; 0}(\alpha \to \lambda )$.

**Figure 2:**
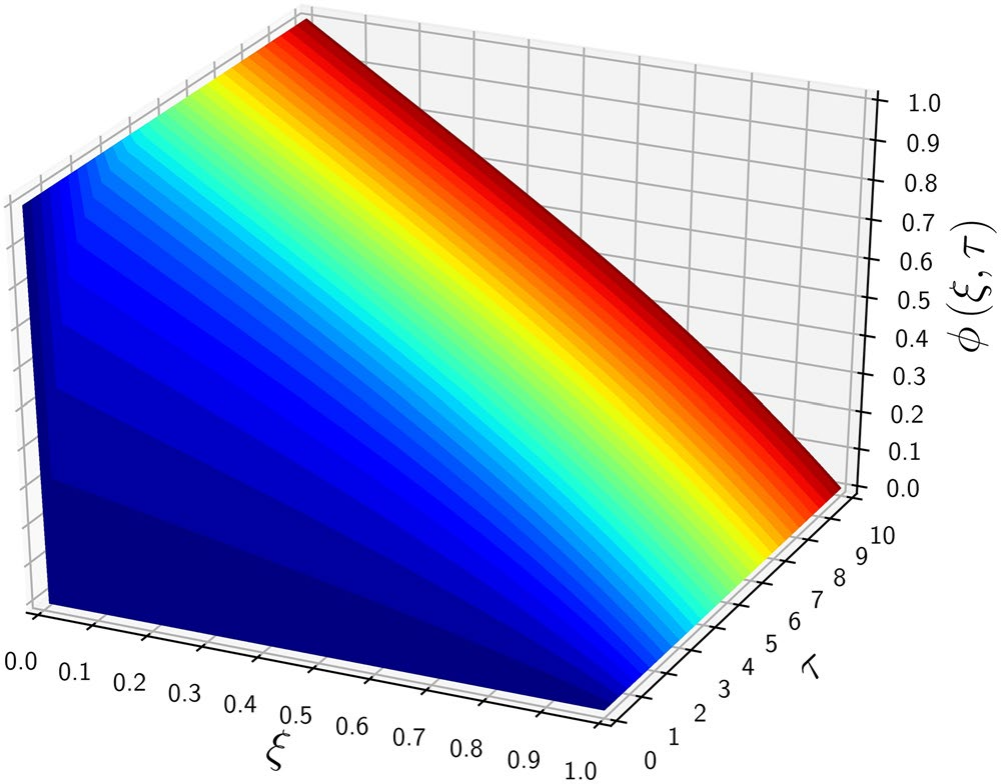
A 3D plot of $\phi$ versus $\tau$ and $\xi$, employing the same parameters used in Fig. 1.

To verify the current analytic method, a specific example of Carslaw’s work^10^ is selected: ${\phi }_{0}=1$, ${\phi }_{1}=0$, ${\mu }_{0}=0$, $\lambda =\mathrm{1/2}$, $\kappa =0$, and $\sigma =0$. A general solution (after the LT) in the absence of a source/sink is equal to the complementary solution of Eq. (41) after the integration parameter $p$ is replaced by a complex variable $z$ (see Method section for details):

3 $$\Phi (\xi ,z)=\frac{{e}^{\lambda \xi }}{z}\frac{\sinh [\beta (1-\xi )]}{\sinh \beta }$$

where $\beta =\sqrt{{\lambda }^{2}+z}$. Equation (3) indicates that $\Phi$ has two singular poles at $z=0$ and $z=-\,{\lambda }^{2}$ (equivalent to $\sinh \beta =0$). The residue of the first pole (at $z\mathrm{=0}$) is equal to the steady-state solution, denoted ${\phi }_{{\rm{ss}}}$:

4 $${\phi }_{{\rm{s}}{\rm{s}}}(\xi )=\mathop{lim}\limits_{z\to 0}[z{e}^{\tau z}\varPhi (\xi ,z)]={e}^{\lambda \xi }\frac{\sinh [\lambda (1-\xi )]}{\sinh \lambda }$$

and the second residue gives an unsteady solution, denoted ${\phi }_{{\rm{tr}}}(\xi ,\tau )$:

5 $${\phi }_{{\rm{t}}{\rm{r}}}(\xi ,\tau )=\mathop{lim}\limits_{z\to -{\lambda }^{2}}[(z+{\lambda }^{2}){e}^{z\tau }\Phi (\xi ,z)]=-{\phi }_{{\rm{s}}{\rm{s}}}(\xi )\,{e}^{-{\lambda }^{2}\tau }$$

as a product of the negative steady-state solution and transient weighting factor, $\exp (-{\lambda }^{2}\tau )$. The sum of Eqs. (4) and (5), i.e., $\phi (\xi ,\tau )={\phi }_{{\rm{s}}{\rm{s}}}(\xi )[1-\exp (-{\lambda }^{2}\tau )]$, is the final solution satisfying the IC, but it does not provide the inlet BC of *ϕ*_0_ = 1 for arbitrary *τ*. Therefore, a proper conditional representation of the full transient solution is

6 $${\phi }_{tr}(\xi ,\tau )=\left\{\begin{array}{ccc}-{\phi }_{ss}(\xi ){e}^{-\tau {\lambda }^{2}} & {\rm{f}}{\rm{o}}{\rm{r}} & 0 < \xi \le 1\\ 1 & {\rm{f}}{\rm{o}}{\rm{r}} & \xi =0\end{array}\right.$$

which resolves the discrepancy of the $\phi (\xi =\mathrm{0,}\,\tau =0)$ value from ${\mu }_{0}=0$ and *ϕ*_0_ = 1. To avoid the above conditional representation, an alternative expression was obtained by using a Fourier series^11^

7 $${\phi }_{{\rm{t}}{\rm{r}}}(0\le \xi \le 1,\,\tau )=-2\pi \lambda {e}^{\lambda \xi }\mathop{\sum }\limits_{n=1}^{{\rm{\infty }}}\,\frac{n\,\sin (n\pi \xi )}{{\lambda }^{2}+{n}^{2}{\pi }^{2}}{e}^{-({\lambda }^{2}+{n}^{2}{\pi }^{2})\tau }$$

as an improved analytic form from the previous work^10^. Figure 1 shows the time-evolution of $\phi$ along $\xi$ from the initial $(\tau =0)$ to the steady states. The initial profile of ${\mu }_{0}=0$ is shown as a horizontal line on the $\xi$ axis, having a discontinuity with the BC of $\phi (\mathrm{0,}\tau )=1$. This concentration jump at the inlet ($\xi =0$) prevails until the steady state is reached. As $\phi$ increases from 0 to the steady-state profile over the entire domain except the boundaries shown in Fig. 2, the dimensionless time scale required to reach the steady state is graphically observed between $\tau =0$ and 10 (of an order of $O(\mathrm{2/}{\lambda }^{2})$). The conditional expression of Eq. (6) has a simpler transient dependence as a product of the steady-state solution and exponential transient decay, but the Fourier series of Eq. (7) resolves the discontinuity of an L-shaped initial concentration profile at the inlet boundary by using an infinite series.

**Figure 3:**
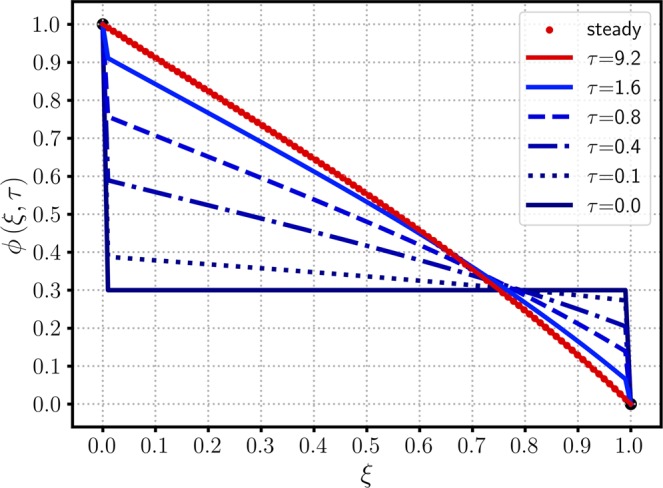
The time-evolution of $\phi (\xi ,\tau )$ for various $\tau$ values versus $\xi$. The same parameters of Fig. 1 are used, except a new exit boundary value of ${\mu }_{0}\mathrm{=0.3}$.

**Figure 4:**
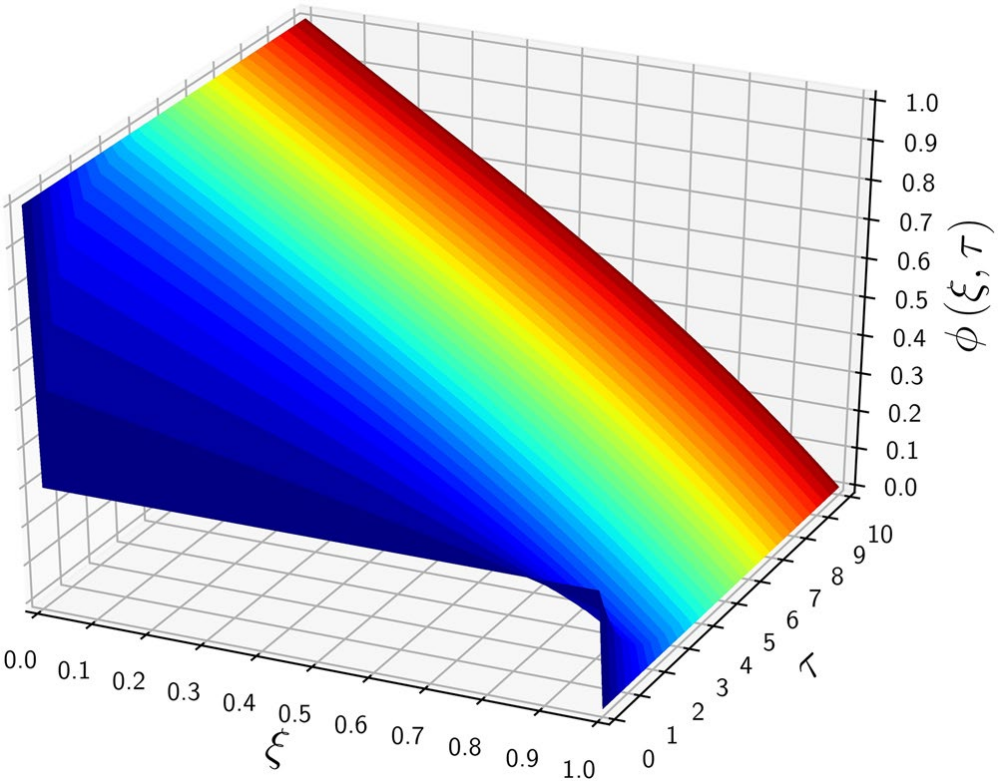
A 3D plot of $\phi$ versus $\tau$ and $\xi$, employing the same parameters used in Fig. 3.

**Figure 5:**
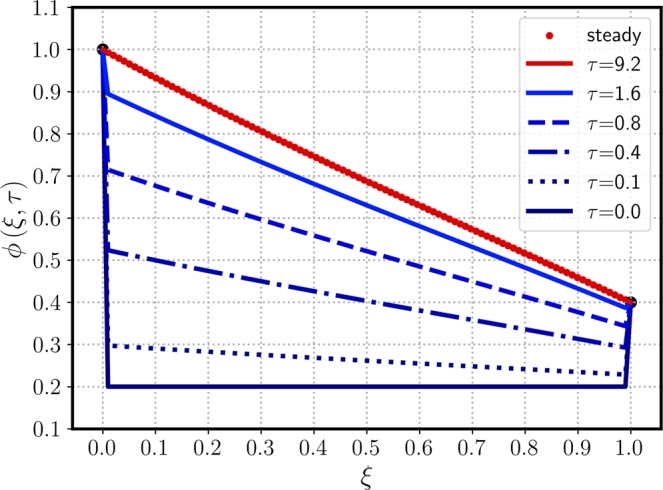
The time-evolution of $\phi (\xi ,\tau )$ from the initial to the steady-state concentration profiles for ${\phi }_{0}\,\mathrm{=\; 1}$, ${\phi }_{1}\,\mathrm{=\; 0.4}$, ${\mu }_{0}\phantom{\rule{ 0.25em}{0ex}}\mathrm{=\; 0.2}$, $\lambda \,\mathrm{=\; 1/2}\,({\rm{Pe}}\,\mathrm{=\; 1})$ and $\kappa \,\mathrm{=\; 0}\,(\alpha \to \lambda )$.

**Figure 6:**
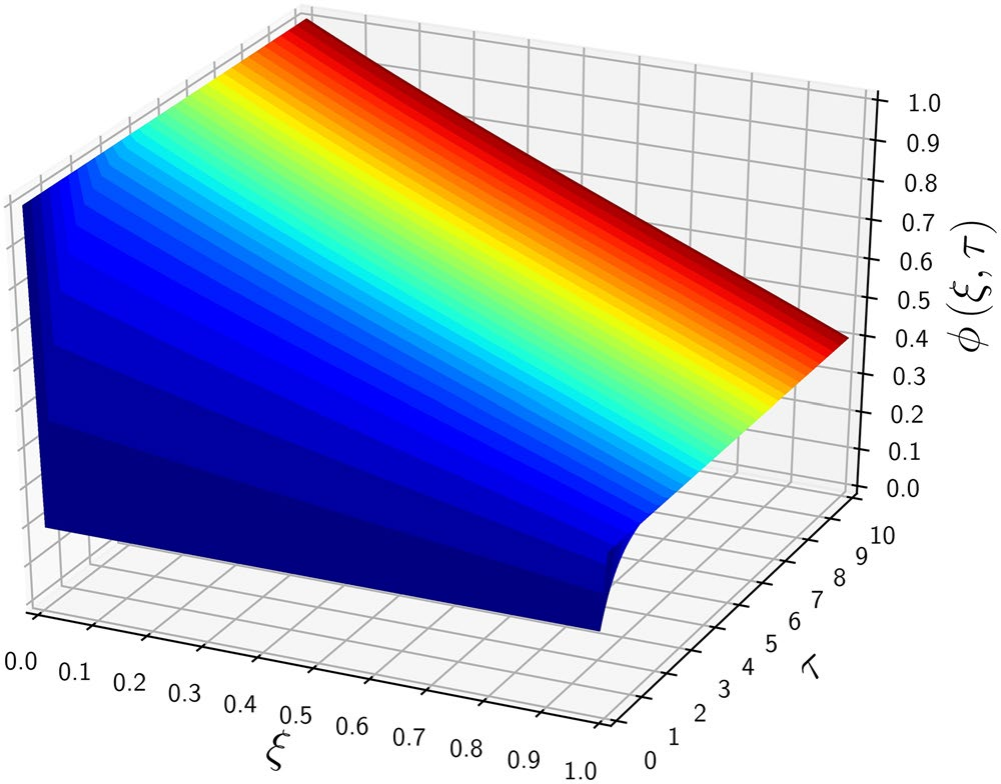
A 3D plot of $\phi$ versus $\tau$ and $\xi$, employing the same parameters used in Fig. 5.

This problem is extended for non-zero values of ${\phi }_{1}$, ${\mu }_{0}$, and $\kappa$, for which the analytic solution is obtained as

8 $$\begin{array}{ccc}\phi (0 < \xi  < 1,\tau ) & = & \frac{{\phi }_{0}\sinh \,(\alpha (1-\xi ))+{\phi }_{1}{e}^{-\lambda }\sinh \,(\alpha \xi )}{\sinh \,\alpha }{e}^{\lambda \xi }[1-{e}^{-{\alpha }^{2}\tau }]\\  &  & -\frac{{\mu }_{0}\sinh \,(\lambda (1-\xi ))+{\mu }_{0}{e}^{-\lambda }\sinh \,(\lambda \xi )}{\sinh \,\lambda }{e}^{\lambda \xi }\,[1-{e}^{-{\lambda }^{2}\tau }\,]{e}^{-\kappa \tau }+{\mu }_{0}{e}^{-{\alpha }^{2}\tau }\end{array}$$

(See the Supplementary Information for detailed derivations.) In Eq. (8), the first term on the right-hand-side represents the spatial variation of $\phi$ from the two boundary values of ${\phi }_{0}$ and ${\phi }_{1}$, which becomes the steady-state solution as $\tau \to \infty$; and the second and third terms show the variation in $\phi (\xi ,\tau )$ originating from the initial concentration ${\mu }_{0}$. At $\tau =0$, the first and second terms disappear and the third term confirms the initial concentration ${\mu }_{0}$. Equation (8) converges analytically to the previous Carslaw case by setting ${\phi }_{0}=1$, ${\phi }_{1}=0$, ${\mu }_{0}=0$, and $\kappa =0$ $(\alpha \to \lambda )$. Figure 3 shows a similar concentration profile to that of Fig. 1 except the initial concentration ${\mu }_{0}=0$ replaced with 0.3, and all other parameters remain the same. Because the time scale to reach the steady state is solely governed by the $\lambda$ and $\kappa$ values, the converging trends of $\phi (\xi ,\tau )$ toward ${\phi }_{{\rm{ss}}}(\xi )$ are similar in Figs. 1 and 3. Due to the same BCs used in Figs. 1 and 3, both cases provide the identical steady-state solution of ${\phi }_{{\rm{ss}}}(\xi )$ of Eq. (4) regardless of IC values. Figure 4 shows a 3D plot of $\phi$ versus $\tau$ and $\phi$, emphasizing the independence of the steady state on the initial conditions. After $\tau$ exceeds around 1.0, the transient solution start converging to that of the steady state. Figure 5 considers a more general case in which the exit boundary value is non-zero, e.g, ${\phi }_{1}=0.4$ where ${\mu }_{0}=0.2$. A finite ${\phi }_{1}$ may have better practicality in heat transfer applications, and therefore it is investigated here to confirm the mathematical robustness of the analytic solution of Eq. (8). In Figs. 5 and 6, the concentration $\phi$ increases from its initial value ${\mu }_{0}=0.2$, meeting the BCs of finite ${\phi }_{0}$ and ${\phi }_{1}$ and reaching the steady state. The time-evolution trend of Fig. 5 is similar to those in Figs. 1 and 3, showing ${\phi }_{{\rm{ss}}}$ monotonically decreasing from the inlet to the outlet. Figure 6 also indicates that $\phi (\xi ,\tau )$ starts converging to the steady state when $\tau$ passes around 1.

The inverse Laplace transform (iLT) is often challenging to obtain both analytically and numerically, owing to the sophisticated nature of singularity identification^9,10^ and numerical sensitivity depending on specific algorithms applied. The uniqueness of the current approach is that the IC is used not only for the LT of the dimensionless governing Eq. (36) but also for the derivation of the unsteady part of the analytic solution without directly performing an iLT.

### Solution for Mixed Dirichlet-Neumann Boundary Conditions

The approximate semi-unbound system is investigated by considering a Neumann-type BC at the outlet (instead of ${\phi }_{1}\,\mathrm{=\; 0}$) such as

9 $$\frac{\partial \phi (\xi \,\mathrm{=\; 1,}\,\tau )}{\partial \xi }={J}_{1}$$

where ${J}_{1}\,\mathrm{=\; 0}$ is set for simplicity, for which the LT is nullified. A set of the inlet-Dirichlet and outlet-Neumann BCs gives a matrix form to obtain coefficients ${B}_{1}$ and ${B}_{2}$ of the complementary part:

10 $$[\begin{array}{cc}1 & 1\\ (\lambda -\beta ){e}^{-\beta } & (\lambda +\beta ){e}^{\beta }\end{array}]\,[\begin{array}{c}{B}_{1}\\ {B}_{2}\end{array}]=[\begin{array}{c}{\phi }_{0}/p\\ 0\end{array}]$$

By substituting ${B}_{1}$ and ${B}_{2}$ into the complementary solution of Eq. (41), one can obtain

11 $$\Phi (\xi ,z)=\frac{\Lambda (\xi ,z)}{z}={\phi }_{0}\,\frac{{e}^{\lambda \xi }}{z}\,\frac{\lambda \,\sinh [\beta (1-\xi )]+\beta \,\cosh [\beta (1-\xi )]}{\lambda \,\sinh \beta +\beta \,\cosh \,\beta }$$

where the steady-state solution is obtained as $\Lambda (\xi ,z)$ at the primary pole $z\,\mathrm{=\; 0}$ (see Method section and Supplementary Information for details), such as

12 $${\phi }_{{\rm{s}}{\rm{s}}}(\xi )={\phi }_{0}{e}^{\lambda \xi }\,\frac{\lambda \,\sinh [\alpha (1-\xi )]+\alpha \,\cosh [\alpha (1-\xi )]}{\lambda \,\sinh \alpha +\alpha \,\cosh \,\alpha }$$

**Figure 7:**
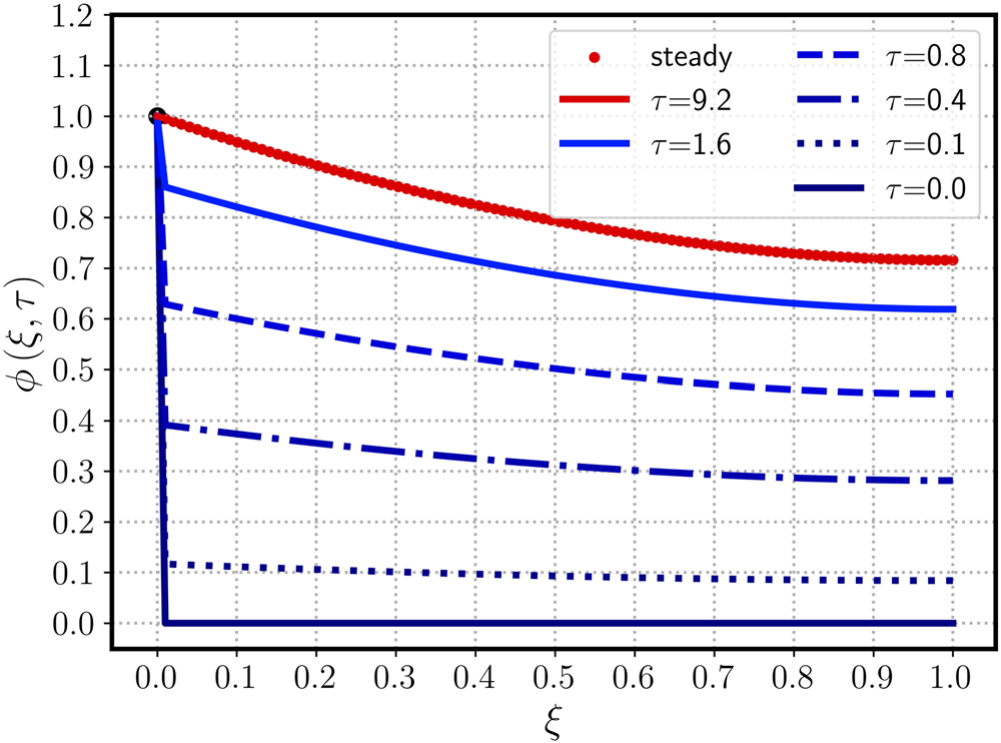
The time-evolution of $\phi (\xi ,\tau )$ from the initial to the steady-state concentration profile for ${\phi }_{0}\,\mathrm{=\; 1}$, $\partial {\phi }_{1}/\partial \xi \,\mathrm{=\; 0}$, ${\mu }_{0}\,\mathrm{=\; 0}$, $\lambda \,\mathrm{=\; 1/2}\,({\rm{Pe}}\,\mathrm{=\; 1})$ and $\kappa \,\mathrm{=\; 1}$.

Then, the iLT is simply written as

13 $$\phi (\xi ,\tau )={\phi }_{ss}(\xi )+lim\,{\rm{R}}{\rm{e}}{\rm{s}}{\rm{i}}{\rm{d}}{\rm{u}}{\rm{e}}\,{\rm{o}}{\rm{f}}\left(\frac{{{\rm{e}}}^{\tau {\rm{z}}}}{{\rm{z}}}\Lambda (\xi ,{\rm{z}})\right)$$

where ${z}_{1}=-\,{\alpha }^{2}$ is the first singularity pole of the transient part that satisfies $\lambda \sinh \,\beta +\beta \cosh \,\beta \,=\,0$. Moreover, by using the zero initial concentration $({\mu }_{0}\,\mathrm{=\; 0})$, one can find

14 $$\mathop{lim}\limits_{z\to {z}_{1}}{\rm{R}}{\rm{e}}{\rm{s}}{\rm{i}}{\rm{d}}{\rm{u}}{\rm{e}}\,{\rm{o}}{\rm{f}}\,[{z}^{-1}\Lambda (\xi ,z)]=-{\phi }_{{\rm{s}}{\rm{s}}}(\xi )$$

and represent the final analytic solution of a conditional form

15 $$\phi (\xi ,\tau )=\{\begin{array}{l}{\phi }_{{\rm{ss}}}(\xi )\cdot [1-{e}^{-{\alpha }^{2}\tau }]\\ 1\end{array}\begin{array}{l}\,{\rm{for}}\,\mathrm{0\;  < }\,\xi \le 1\\ \,{\rm{for}}\,\xi \,\mathrm{=\; 0}\end{array}$$

which satisfies the outlet BC of the zero asymptotic flux $\frac{\partial }{\partial \xi }{\phi }_{{\rm{ss}}}(1)\,\mathrm{=\; 0}$. The denominator of Eq. (12) increases with $\kappa$ (through $\alpha$) having a fixed $\lambda$, thus confirming that the steady – state concentration ${\phi }_{{\rm{ss}}}(\xi )$ reduces for high reactivity $\kappa$. The time scale to reach the steady state is of an order of $O({\alpha }^{-2})$ not only for this zero outlet flux case but also for other previous cases. A full solution with non-zero flux ${J}_{1}$ and constant ${\mu }_{0}$ is calculated as follows

16 $$\phi (\xi ,\tau )=[{J}_{1}{e}^{-\lambda }h(\xi )+{\phi }_{0}g(\xi )]{e}^{\lambda \xi }(1-{e}^{-{\alpha }^{2}\tau })+{\mu }_{0}[1-g(\xi ){e}^{\lambda \xi }(1-{e}^{-{\lambda }^{2}\tau })]{e}^{-\kappa \tau }$$

where

17 $$g(\xi ,\alpha ,\lambda )=\frac{\lambda \,\sinh [\alpha (1-\xi )]+\alpha \,\cosh [\alpha (1-\xi )]}{\lambda \,\sinh \,\alpha +\alpha \,\cosh \,\alpha }$$

18 $$h(\xi ,\alpha ,\lambda )=\frac{\sinh \,\alpha \xi }{\lambda \,\sinh \alpha +\alpha \,\cosh \alpha }$$

whose detailed derivation can be found in the Supplementary Information. Figure 7 shows the unsteady profiles of $\phi (\xi ,\tau )$ having the inlet – Dirichlet $({\phi }_{0}\,\mathrm{=\; 1})$ and outlet – Neumann $({J}_{1}\,\mathrm{=\; 0})$ BCs. The time-evolution trend of Fig. 7 is similar to those of Figs. 1, 3 and and 5, except for the increasing concentration at the outlet with time to satisfy the constant flux ${J}_{1}\,\mathrm{=\; 0}$. The outlet boundary value of Fig. 7 at the steady state for arbitrary $\kappa$ is

19 $${\phi }_{{\rm{s}}{\rm{s}}}(\xi \,=\,1)\,=\,\frac{{\phi }_{0}\alpha {e}^{\lambda }}{\lambda \,\sinh \,\alpha +\alpha \,\cosh \,\alpha }$$

which converges to various interesting cases such as

20 $${\phi }_{{\rm{ss}}}(\xi \,\mathrm{=\; 1,}\kappa \,\mathrm{=\; 0})\,=\,{\phi }_{0}$$

21 $${\phi }_{{\rm{ss}}}(\xi \,\mathrm{=\; 1,}\lambda \to \infty )\,=\,{\phi }_{0}$$

22 $${\phi }_{{\rm{s}}{\rm{s}}}(\xi \,=\,1,\lambda \,=\,0)\,=\,\frac{{\phi }_{0}}{\cosh \,\kappa }$$

of which physical meanings are as follows. The concentration $\phi$ is maintained at the level of the inlet concentration ${\phi }_{0}$ if the reaction is absent (of Eq. (20)) or the convection is predominant over diffusion and reaction (of Eq. (21)). The reaction process significantly decreases the outlet concentration due to the zero-flux in the absence of convection (of Eq. (22)). The influence of the exit BC on the evolution of $\phi$ to the steady-state is shown in Fig. 8. As the outlet concentration is not forcefully bounded by constant ${\phi }_{1}$, the overall profile $\phi$ rapidly increases from zero within a time interval, $0 < \tau \le 1$, instantaneously satisfying the zero outlet-flux.

**Figure 8:**
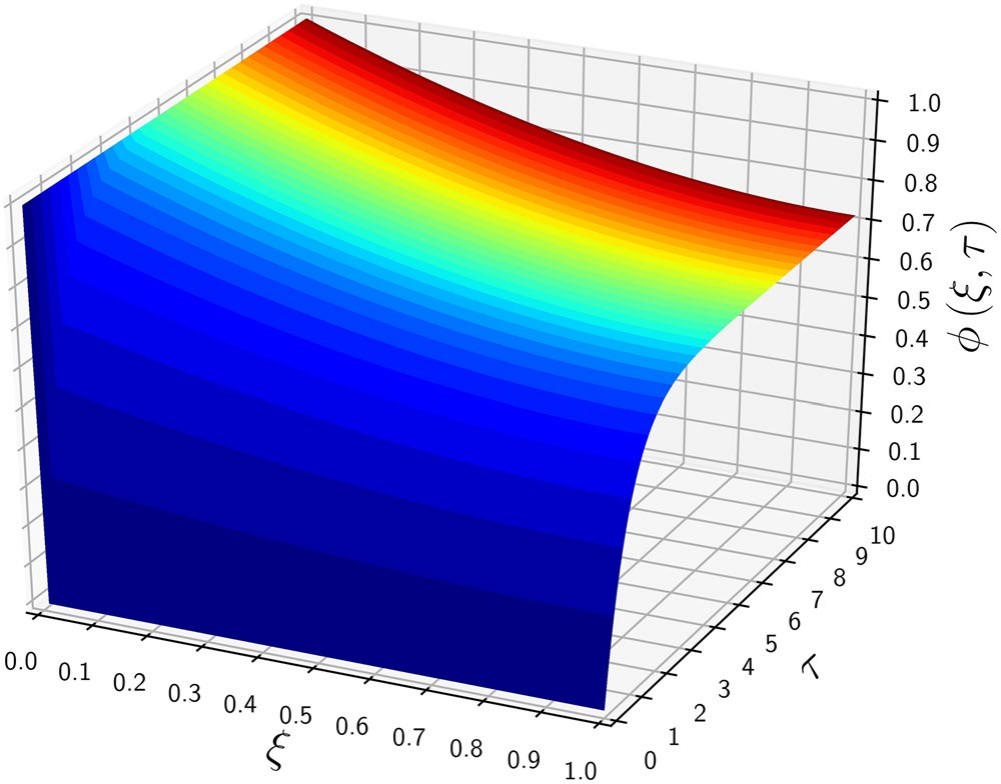
A 3D plot of $\phi$ versus $\tau$ and $\xi$, employing the same parameters used in Fig. 7.

### Solution for a full CDRS equation with spatially varying source function

As discussed above, the analytic solution of the CDRS equation is subject to finding the steady-state solution corresponding to $\Lambda (\xi ,z\,=\,0)$ and calculating the unsteady part by using the IC. This argument seems to be, however, restricted to the governing CDRS equation with constant initial concentration without any source or sink functions. In this section, a sophisticated case is discussed for $\lambda =-\mathrm{1/2}$ and $\kappa =-1$, taken from Zhong *et al*.’s work^12^ that used a spatially varying source function of

23 $$\sigma (\xi )\,=\,{e}^{-a\xi }\,\sin \,(b\xi )$$

where $a\,=\,\pi \mathrm{/2}$ and $b\,\mathrm{=\; 5}\pi$. Substitution of Eq. (23) into (42) with BCs of ${\phi }_{0}\,=\,{\phi }_{1}\,\mathrm{=\; 0}$ gives

24 $$\Lambda (\xi ,z)=\frac{{\Phi }_{P}(\xi ,z)}{z}={e}^{-a\xi }\frac{\sin (b\xi -\theta )}{|Z|}={e}^{-a\xi }\frac{[{b}^{2}-{\tilde{a}}^{2}-{\left(\frac{\gamma }{2}\right)}^{2}]\sin \,b\xi -2b\tilde{a}\cdot \cos b\xi }{\left({\tilde{a}}^{2}+{\left(b+\frac{1}{2}\gamma \right)}^{2}\right)\left({\tilde{a}}^{2}+{\left(b-\frac{1}{2}\gamma \right)}^{2}\right)}$$

where

25 $$Z\,=\,\sqrt{{\left({b}^{2}-{\tilde{a}}^{2}-{\left(\frac{\gamma }{2}\right)}^{2}\right)}^{2}+{(2\tilde{a}b)}^{2}}$$

26 $$\theta \,=\,{\tan }^{-1}\left(\frac{2\mathop{a}\limits^{ \sim }b}{{b}^{2}-{\mathop{a}\limits^{ \sim }}^{2}-{\left(\frac{\gamma }{2}\right)}^{2}}\right)$$

having $\tilde{a}\,=\,a-\frac{1}{2}$ and $\gamma \,\mathrm{=\; 2}\sqrt{\frac{3}{4}-z}$. Note that the zero BCs relate the particular and the complementary solutions because the combined form should satisfy required conditions. Following the one-sided LT described in the Method section, the particular steady-state solution is obtained as ${\phi }_{P,{\rm{s}}{\rm{s}}}(\xi )\,=\,\Lambda (\xi ,z\,=\,0)$ by replacing $\gamma$ with $\sqrt{3}$. The BCs, ${\phi }_{0}\,=\,0$ and ${\phi }_{1}\,=\,0$, determine the coefficients of the complementary solution

27 $${\varPhi }_{C}(\xi ,z)\,=\,{B}_{1}{e}^{{\lambda }_{m}\xi }+{B}_{2}{e}^{{\lambda }_{p}\xi }$$

with the two coefficients determined as

28 $${B}_{1}\,=\,\frac{{\varPhi }_{P}(1)-{e}^{{\lambda }_{p}}\varPhi (0)}{2{e}^{\lambda }\sinh \,(\beta )}$$

29 $${B}_{2}\,=\,\frac{{e}^{{\lambda }_{m}}\,{\varPhi }_{P}(0)-\varPhi (1)}{2{e}^{\lambda }\sinh \,(\beta )}$$

where ${\lambda }_{m}\,=\,\lambda -\beta$, ${\lambda }_{p}\,=\,\lambda +\beta$, and $\beta \,=\,\sqrt{z-\frac{3}{4}}$. The iLT of ${\Phi }_{C}$ will produce the complementary solution ${\phi }_{C,{\rm{ss}}}$ in real space. The iLT of the full solution $\Phi (\xi ,z)\,=\,{\Phi }_{C}(\xi ,z)+{\Phi }_{P}(\xi ,z)$ gives:

30 $${\phi }_{{\rm{s}}{\rm{s}}}(\xi )={e}^{-\xi /2}\left[{b}_{1}\sin \left(\frac{\sqrt{3}}{2}\xi \right)+{b}_{2}\cos \left(\frac{\sqrt{3}}{2}\xi \right)\right]+\frac{{e}^{-a\xi }\sin (b\xi -{\theta }_{0})}{|{Z}_{0}|}$$

using

31 $${b}_{1}\,=\,\frac{\sin {\theta }_{0}}{|{Z}_{0}|}$$

32 $${b}_{2}=-\frac{\sin {\theta }_{0}\,cos(\frac{\sqrt{3}}{2})+{e}^{-\mathop{a}\limits^{ \sim }}\sin (b-{\theta }_{0})}{|{Z}_{0}|}$$

where subscript 0 of ${\theta }_{0}$ and ${Z}_{0}$ indicates ${\gamma }_{0}\,=\,\gamma (z\,\mathrm{=\; 0})$ is used. As a proof of the current method, the steady-state solution of Eq. (30) is independently derived using the standard mathematical technique of integral factors. (See Supplementary Information for details.) The full steady state solution is then represented as

33 $${\phi }_{{\rm{ss}}}(\xi )\,=\,\{\begin{array}{l}{\phi }_{C,{\rm{ss}}}(\xi )+{\phi }_{P,{\rm{ss}}}(\xi )\\ 0\end{array}\begin{array}{l}\,{\rm{for}}\,\mathrm{0\;  < }\,\xi \,\mathrm{ < \; 1}\\ \,{\rm{for}}\,\xi \,\mathrm{=\; 0,\; 1}\end{array}$$

Possible negative values of $\phi$ do not indicate that the solution ${\phi }_{{\rm{ss}}}$ has unacceptable physical meaning, but instead indicate that it is ascribed to the zero initial concentration. Although the source function $\sigma$ does not explicitly depend on $\phi$, finite $\kappa$ (regardless of its sign) does not allow translational invariance of $\phi$; i.e., $\phi$ and $\phi +\varepsilon$, where $\varepsilon$ is a constant, are not governed by the identical transport equation. The singularity of Eq. (24) occurs at $z=-\left({b}^{2}-\frac{3}{4}-{\tilde{a}}^{2}\right)\pm i(2b\tilde{a})$, which is found by setting the denominator of $\Phi$ to zero. Then, the coefficient of the $\sin (b\xi )$ term becomes

34 $${b}^{2}-{\mathop{a}\limits^{ \sim }}^{2}-{\left(\frac{\gamma }{2}\right)}^{2}\,=\mp 2i\mathop{a}\limits^{ \sim }b$$

and therefore the time-dependence is ${e}^{-({b}^{2}-{\mathop{a}\limits^{ \sim }}^{2}-\frac{3}{4})\tau }\sin (2\mathop{a}\limits^{ \sim }b\tau )$ weighted to the steady-state solution, thus yielding the final solution of

35 $$\phi (\xi ,\tau )\,=\,\{\begin{array}{c}{\phi }_{{\rm{s}}{\rm{s}}}(\xi )[1-{e}^{-({b}^{2}-{\mathop{a}\limits^{ \sim }}^{2}-\frac{3}{4})\tau }\sin (2\mathop{a}\limits^{ \sim }b\tau )]\\ 0\end{array}\begin{array}{c}\,{\rm{f}}{\rm{o}}{\rm{r}}\,\tau \, > \,0\,{\rm{a}}{\rm{n}}{\rm{d}}\,0\, < \,\xi \, < \,1\\ \,{\rm{f}}{\rm{o}}{\rm{r}}\,\tau \,=\,0\,{\rm{o}}{\rm{r}}\,\xi \,=\,0,1\end{array}$$

**Figure 9:**
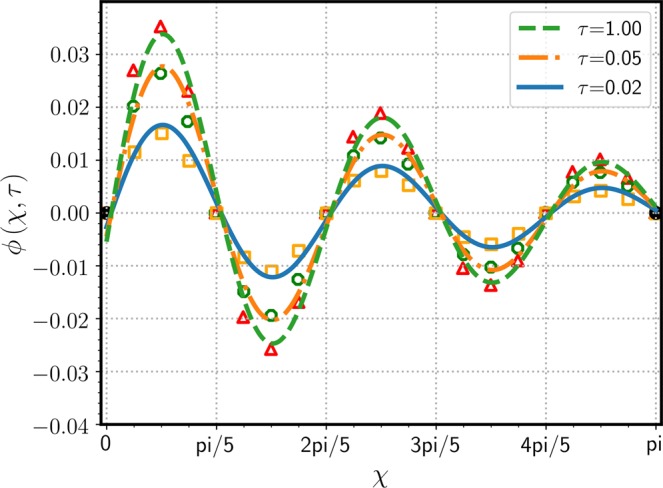
Variation of $\phi (\chi ,\tau )$ with respect to $\xi$ at three time steps of $\tau \,=\,$ 0.02 (▫), 0.05 (○), and 1 ($\bigtriangleup$). For direct comparison between the analytic and numerical results, values are taken from Zhong *et al*.’s work^12^(${\mu }_{0}\,\mathrm{=\; 0}$, ${\phi }_{0}\,\mathrm{=\; 0}$, and ${\phi }_{1}\,\mathrm{=\; 0}$), where $\chi \,=\,\pi \xi$ is used for the domain length $L\,=\,\pi$.

**Figure 10:**
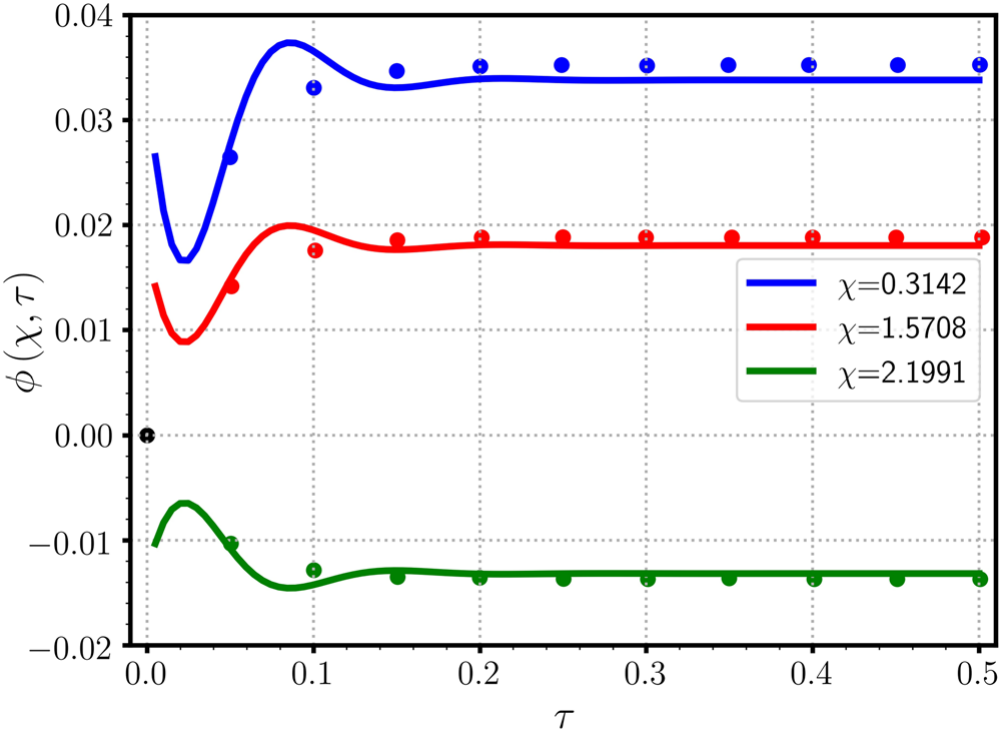
Variation of $\phi (\chi ,\tau )$ with respect to $\tau$ at three fixed locations of $\chi \,=\,$ 0.3142, 1.5708, and 2.1991. Parameters used were same as those employed in Fig. 9.

**Figure 11:**
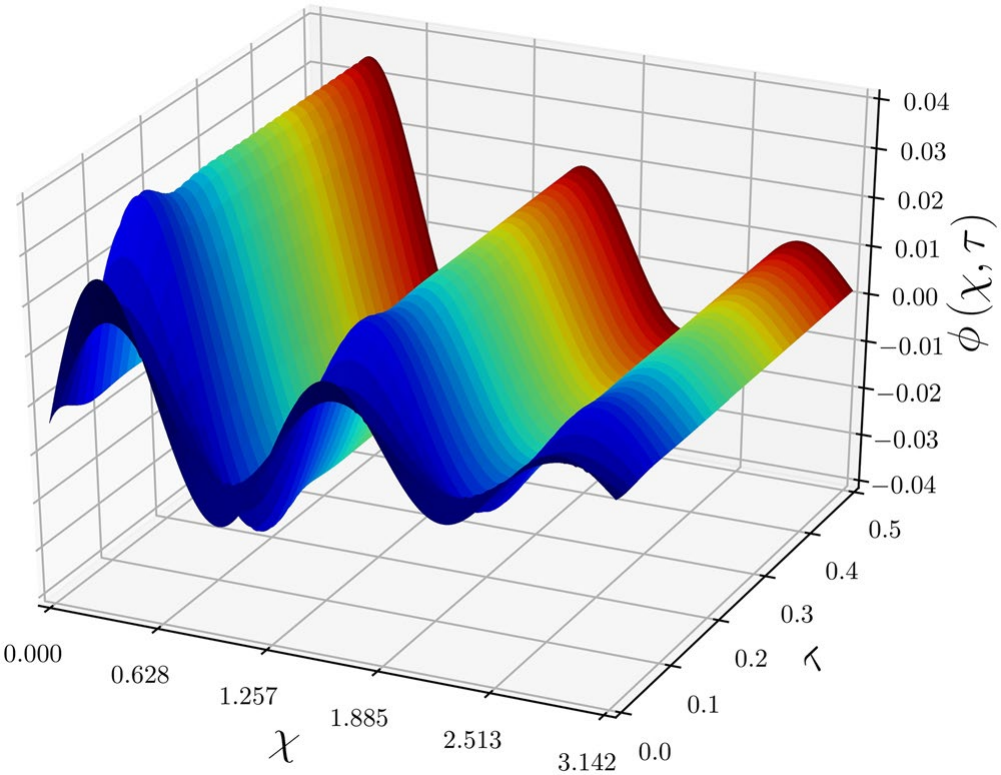
A 3D plot of $\phi (\chi ,\tau )$, representing the time-evolution of work by Zhong *et al*.^12^ using with ${\mu }_{0}\,\mathrm{=\; 0}$, ${\phi }_{0}\,\mathrm{=\; 0}$, and ${\phi }_{1}\,\mathrm{=\; 0}$.

Figure 9 shows reasonably good agreement between the present theoretical work and Zhong *et al*.’s simulational results^12^, which predict spatial variation of $\phi (\xi ,\tau )$ (satisfying the BCs of zero concentrations ${\phi }_{0}\,=\,{\phi }_{1}\,\mathrm{=\; 0}$) at three different instances. As Zhong *et al*. set the domain length $L\,=\,\pi$, a new axis is defined as $\chi \,=\,\pi \xi$ and used for Figs. 9–11. The initial concentration ${\mu }_{0}\,\mathrm{=\; 0}$ is superceded on the horizontal axis of Fig. 9. The numerical solution for $\phi$ seems to reach zero at $\chi \,=\,n\pi \mathrm{/5}$, which was expected by the functional form of $\sigma$ of Eq. (23), but the analytic solution of Eq. (30) clearly indicates that a phase shifts as much as ${\theta }_{0}$, which is calculated as 0.1365 in radians. At the first and second extrema at $\chi \,=\,\pi \mathrm{/10}$ and $3\pi \mathrm{/10}$, respectively, the numerical solution deviates from the analytic solution, especially for $\tau \,\mathrm{=\; 1}$. Although the theoretical and simulational results show small discrepancies, it is worth noting that the differences are dominant where $\sigma (\xi )$ decreases exponentially with sinusoidal fluctuations. Figure 10 shows variation of $\phi (\xi ,\tau )$ with respect to $\tau$ at three different locations. Both solutions show the rapid convergence of $\phi$ toward its state-state value after $\tau \,\mathrm{ > \; 0.2}$. The rapidly fluctuating phenomena in the initial state $(\tau \,\mathrm{ < \; 0.1})$ were not captured by the numerical solution, mostly because the time interval was not short enough. Zhong *et al*. indicated that slight mismatches between FEM and SGM results were due to unequal time-step sizes, however, specific grid size or time interval were not reported. In the presence of the source function such as $\sigma$ of Eq. (23) decreasing stiffly from $\xi \,\mathrm{=\; 0}$ for $\tau \,\mathrm{ > \; 0}$, constant grid size may not be the best practice; instead, a better approach can be a variable grid size being inversely proportional to the concentration gradient in magnitude. Figure 11 clearly shows the initial fluctuation of $\phi$ with respect to $\tau$ along the $\chi$ direction, which was not visually observed in the numerical work of Zhong *et al*.^12^ in Fig. 9. The spatial variation of $\phi$ is primarily due to the variation pattern of the source function $\sigma$ and the transient behavior is ascribed to Eq. (35) of diminishing sinusoidal variation. Figure11 also implies the necessity of the variable grid-steps (in $\xi$ direction) based on the concentration gradient.

A more complex form of a source function can be represented using multiple terms or a Fourier series. As the general solution for a source function of Eq. (23) is obtained in this study, it is straightforward to extend the current method for cases of functionally sophisticated source terms as long as the complexity of the functional forms provide no more than one non-zero singularity pole. In other words, because a linear combination of $\sigma (\xi )$ and ${\mu }_{I}(\xi )$ is included as an integrand to obtain $I$ $(\xi ,p)$ and $\varPhi (\xi ,p)$, mathematical procedures to obtain the final analytic solution $\phi$ of the CDRS equations are straightforward if $\sigma$ and ${\mu }_{I}$ are expressed with sinusoidal functions and/or infinite Fourier series.

## Method

### Solution Procedure of a CDRS Equation

To solve the dimensionless governing Eq. (2), the IC is given as $\phi (\xi \mathrm{,0})\,=\,{\mu }_{I}(\xi )$, and BCs are paired as Dirichlet-Dirichlet or Dirichlet-Neumann combinations as follows. First, two Dirichlet BCs are specified as $\phi ({\xi }_{0},\tau )\,=\,{\phi }_{0}(\tau )$ and $\phi ({\xi }_{1},\tau )\,=\,{\phi }_{1}(\tau )$. A frequently used example consists of ${\phi }_{0}\,\mathrm{=\; 1}$ and ${\phi }_{1}\,\mathrm{=\; 0}$, indicating a specified inlet concentration scaled by ${C}_{\infty }$ and the perfect sink of mass at the outlet, respectively. Second, the outlet condition can be of the Neumann-type, by using a concentration gradient, often representing the zero flux of ${[\partial \phi (\xi ,\tau )/\partial \xi ]}_{\xi \to {\xi }_{1}}\,\mathrm{=\; 0}$ in a semi-infinite channel $(L\to \infty )$. Then, Eq. (2) is rewritten as

36 $$[{{\mathscr{D}}}_{\xi }-{\lambda }_{m}]\,[{{\mathscr{D}}}_{\xi }-{\lambda }_{p}]\Phi =-\Omega (\xi )$$

where $${{\mathscr{D}}}_{\xi }\,=\,\frac{\partial }{\partial \xi }$$, $${\lambda }_{m}\,=\,\lambda -\beta $$, $${\lambda }_{p}\,=\,\lambda +\beta $$, $$\beta \,=\,\sqrt{{\alpha }^{2}+p}$$, $${\alpha }^{2}\,=\,{\lambda }^{2}+\kappa $$, $$\Omega (\xi )\,=\,{\mu }_{I}(\xi )+{p}^{-1}\sigma (\xi )$$ and

37 $$\Phi (\xi ,p):={\mathcal{L}}[\phi (\xi ,\tau )](p)\,=\,{\int }_{0}^{{\rm{\infty }}}{e}^{-p\tau }\phi (\xi ,\tau ){\rm{d}}\tau $$

as the LT of the dimensionless concentration $\phi (\xi ,\tau )$. In Eq. (36), $\Omega (\xi )$ plays a role of a source/sink term in the Laplace-transformed space. A new function is defined for simplicity as follows:

38 $${\Phi }^{\dagger }\equiv [{{\mathscr{D}}}_{\xi }-{\lambda }_{p}]\Phi \,=\,{e}^{{\lambda }_{p}\xi }{{\mathscr{D}}}_{\xi }(\Phi {e}^{-{\lambda }_{p}\xi })$$

to rewrite Eq. (36) as

39 $$[{{\mathscr{D}}}_{\xi }-{\lambda }_{m}]{\Phi }^{\dagger }\,=\,{e}^{-{\lambda }_{m}\xi }{{\mathscr{D}}}_{\xi }({\Phi }^{\dagger }{e}^{{\lambda }_{m}\xi })=-\Omega (\xi )$$

of which the general solution for ${\varPhi }^{\dagger }$ is

40 $${\Phi }^{\dagger }(\xi )\,=\,{e}^{{\lambda }_{m}\xi }[{B}_{1}-\int {\rm{d}}\eta \,\Omega (\eta ){e}^{-{\lambda }_{m}\eta }]$$

Substitution of Eq. (40) into (39) gives the general solution $\varPhi (\xi ,p)$ as a superposition of the complementary solution ${\varPhi }_{c}(\xi ,p)$ and the particular solution ${\varPhi }_{p}(\xi ,p)$ such that

41 $${\Phi }_{C}(\xi ,p)\,=\,{B}_{1}{e}^{{\lambda }_{m}\xi }+{B}_{2}{e}^{{\lambda }_{p}\xi }\,=\,{e}^{\lambda \xi }({B}_{1}{e}^{-\beta \xi }+{B}_{2}{e}^{\beta \xi })$$

42 $${\varPhi }_{P}(\xi ,p)=-{e}^{{\lambda }_{p}\xi }\int {\rm{d}}\chi {e}^{({\lambda }_{m}-{\lambda }_{p})\chi }\,I(\chi )$$

43 $$\Phi (\xi ,p)\,=\,{\Phi }_{C}(\xi ,p)+{\Phi }_{P}(\xi ,p)$$

where

44 $$I(\chi )\,=\,\int {\rm{d}}\eta {e}^{-{\lambda }_{m}\eta }\,\Omega (\eta )$$

and ${B}_{1}$ and ${B}_{2}$ are unknown constants (from the integrals) to be determined using BCs. If $\Omega (\xi )$ is a constant, denoted as ${\Omega }_{0}$, then the particular solution is simplified to

45 $${\Phi }_{P}(\xi ,p)=-\frac{{\Omega }_{0}}{{\lambda }_{m}{\lambda }_{p}}\,=\,\frac{{\Omega }_{0}}{\kappa +p}$$

Derivatives of the complementary and particular solutions are calculated as follows to use the Neumann BCs:

46 $${\Phi }_{C}^{{\rm{{\prime} }}}(\xi ,p)\,=\,{B}_{1}{\lambda }_{m}{e}^{{\lambda }_{m}\xi }+{B}_{2}{\lambda }_{p}{e}^{{\lambda }_{p}\xi }$$

47 $${\Phi }_{P}^{{\rm{{\prime} }}}(\xi ,p)=-[{\lambda }_{p}{\Phi }_{P}(\xi ,p)+{e}^{{\lambda }_{m}\xi }\,I(\xi )]$$

Of note, in Eqs. (42) and (44), the evaluation ${\Phi }_{P}(\xi ,p)$ and $I(\chi )$ will be straightforward if $\Omega (\xi )$ is given as constant, exponential, or sinusoidal functions. A sophisticated function form of ${\mu }_{I}(\xi )$ or $\sigma (\xi )$ can be described by using a Fourier series. If a BC is given as a Dirichlet-type constant ($\phi (\xi \,=\,{\xi }_{i},\tau )\,=\,{\phi }_{i}$ for $i\,\mathrm{=\; 0}$ or 1), the LT of the BC is obtained by using Eq. (43) as

48 $${B}_{1}{e}^{{\lambda }_{m}{\xi }_{i}}+{B}_{2}{e}^{{\lambda }_{p}{\xi }_{i}}\,=\,\frac{{\phi }_{i}}{p}-{\Phi }_{P}({\xi }_{i},p)$$

In contrast, if a zero-flux BC is assigned at $\xi \,=\,{\xi }_{j}$ for $j\,\mathrm{=\; 0,1}$, its LT is calculated by using Eqs. (46) and (47)

49 $${B}_{1}{\lambda }_{m}{e}^{{\lambda }_{m}{\xi }_{j}}+{B}_{2}{\lambda }_{p}{e}^{{\lambda }_{p}{\xi }_{j}}\,=\,{e}^{{\lambda }_{m}{\xi }_{j}}\,I({\xi }_{j})+p{\Phi }_{P}({\xi }_{j},p)$$

Having two BCs, one can use the iLT in principle to obtain the transient $\phi (\xi ,\tau )$ in real space:

50 $$\phi (\xi ,\tau )\,=\,\frac{1}{2\pi i}{\int }_{c-i{\rm{\infty }}}^{c+i{\rm{\infty }}}{e}^{\tau z}\Phi (\xi ,z){\rm{d}}z\,=\,\frac{1}{2\pi i}{\int }_{c-i{\rm{\infty }}}^{c+i{\rm{\infty }}}{e}^{\tau z}\frac{\Lambda (\xi ,z)}{z}{\rm{d}}z$$

where the real variable $p$ of Eq. (37) is replaced by a complex variable $z$, and $c$ is a constant greater than the real part of the singularities of the integrand $\varPhi$. One can write $\Phi (\xi ,z)\,=\,\Lambda (\xi ,z)/z$, where $\Lambda (\xi ,z)$ is finite at $z\,\mathrm{=\; 0}$, and its residue in the iLT provides the steady-state solution ${\phi }_{{\rm{ss}}}(\xi )$:

51 $$\mathop{lim}\limits_{z\to 0}\,z[{e}^{\tau z}\frac{\Lambda (\xi ,z)}{z}]\,=\,\Lambda (\xi ,0)\,=\,{\phi }_{{\rm{s}}s}(\xi )$$

because the transient term ${e}^{\tau z}$ disappears by setting $z\,\mathrm{=\; 0}$. By combining Eq. (50) at $\tau \,\mathrm{=\; 0}$ and Eq. (51), one can generally write

52 $$\phi (\xi ,\tau )\,=\,\varLambda (\xi ,0)+\sum _{k,{z}_{k}\ne 0}{\rm{R}}{\rm{e}}{\rm{s}}{\rm{i}}{\rm{d}}{\rm{u}}{\rm{e}}\,[{e}^{\tau {z}_{k}}\Phi (\xi ,{z}_{k})]$$

where $\Lambda (\xi ,0)$ is at the primary pole of ${z}_{0}\,\mathrm{=\; 0}$ and ${z}_{k}$ is the pole of order $k\,(\ge 1)$ of $\Phi (\xi ,z)$ function. Note that ${z}_{0}\,\mathrm{=\; 0}$ is one of branch points of function $\Phi (\xi ,{z}_{k})$, but not that of $\varLambda (\xi \mathrm{,0})$. If the primary pole does not exist, the steady-state solution $\varLambda (\xi \mathrm{,0})$ is absent due to the reaction-originated mass transfer, such as a batch reaction in a confined space. In the batch system, the concentration $\phi (\xi ,\tau )$ decreases until it reaches zero due to the reactive mass conversion. (See the Supplementary Information for detailed discussions.) Interestingly, if $\Phi (\xi ,z)$ function has only two poles at $z\,=\,{z}_{0}\,\mathrm{=\; 0}$ and $z\,=\,{z}_{1}$, then Eq. (52) is simplified to

53 $$\phi (\xi ,\tau )\,=\,\varLambda (\xi ,0)+{\rm{R}}{\rm{e}}{\rm{s}}{\rm{i}}{\rm{d}}{\rm{u}}{\rm{e}}\,[\Phi (\xi ,{z}_{1})]\cdot {e}^{\tau {z}_{1}}$$

where calculation of ${\rm{R}}{\rm{e}}{\rm{s}}{\rm{i}}{\rm{d}}{\rm{u}}{\rm{e}}\,[\varPhi (\xi ,{z}_{1})]$ requires an iLT, which can be avoided by applying the IC to Eq. (53):

54 $${\rm{R}}{\rm{e}}{\rm{s}}{\rm{i}}{\rm{d}}{\rm{u}}{\rm{e}}\,[\Phi (\xi ,{z}_{1})]\,=\,{\mu }_{I}(\xi )-{\phi }_{{\rm{s}}s}(\xi )$$

Then, one can conclude that if a LT of concentration $\phi (\xi ,\tau )$ has only one non-zero singularity pole, contributing to the transient behavior, then $\phi (\xi ,\tau )$ is equal to the steady-state solution plus a transient function multiplied by the difference between the initial and the steady-state concentrations, which is conceptually written using Eq. (50) as

55 $$\phi (\xi ,\tau )\,=\,{\phi }_{{\rm{s}}s}(\xi )+[{\mu }_{I}(\xi )-{\phi }_{{\rm{s}}s}(\xi )]\cdot \exp [{\rm{\Re }}({z}_{1})\tau ]$$

where $\Re ({z}_{1})$ is the real part of the imaginary pole ${z}_{1}$. On note, Eq. (52) is obtained by applying the IC, which is equivalent to, and much more convenient than, performing the iLT. If $\varPhi (\xi ,z)$ has $N\,(\ge 2)$ non-zero singularities poles, then the number of required iLTs reduces to $N-1$ by replacing one of the transforms (possibly, the most challenging one) by applying the IC. This method is quite useful if a single-pole integrand of the iLT is quite challenging or not found in reference tables^13–15^.

In addition, numerical iLT, as subjected to integration algorithms and arbitrary determination of $c$ in Eq. (50), were investigated by multiple researchers^16–23^ to minimize effects of complex-domain setting for the integration. Numerical accuracy of the LT was often evaluated by applying a Tauberian theorem:^24,25^ if an arbitrary function $g(t)$ is measurable and bounded on $\mathrm{[0,}\infty )$, so that its LT

56 $$G(z)\,=\, {\mathcal L} [g(t)](z)\,=\,{\int }_{0}^{\infty }g(t){e}^{-zt}{\rm{d}}t,\,z\,=\,x+iy$$

is well-defined and analytic along the positive real axis $\{x\,=\,\Re (z)\mathrm{ > 0\}}$ and $g(t)$ has an analytic extension to the open interval $(-iR,+iR)$ of the imaginary axis, then

57 $$\mathop{lim}\limits_{T\to \infty }\,sup|G(0)-{\int }_{0}^{T}g(t){\rm{d}}t|\le \frac{2M}{R}$$

for every choice of $R$, where

58 $$M\,=\,\mathop{sup}\limits_{t\mathrm{ > 0}}|g(t)|$$

and $sup$ indicates the supremum. This Tauberian theorem for the LT indirectly supports the current derivation to obtain the steady state solution as both limits of $T\to \infty$ and $R\to \infty$ are analytically implemented. In the original work of Talbot^18^, the Bromwich integral^26^ representation was used to transform the integration path into a parabolic parameterized curve that give fast convergence. An improved algorithm – at least twice faster than Talbot’s work – was proposed by Trefethen *et al*.^27^ with fewer parameters, which are more difficult to compute. A universal observation in the iLT to solve the parabolic partial differential equation such as CDRS equation is as follows: first, numerical algorithms for the iLT are available in the literature, but results are subject to specific parameters to be determined; second, as the iLT deals with theoretically infinite imaginary domain, the numerical accuracy cannot be improved by simply increasing the number of divisions of a real-space integration; and third, a numerical result of the iLT is hard to validate unless it is compared with numerical solutions independently obtained using different methods.

The current method is uniquely applicable to solving the CDRS equation – especially when the non-trivial steady state exists – of which mathematical generality needs to be further studied rigorously. Applications of the current method to conventional examples successfully reproduced analytical and numerical results found in literature, especially for cases that the initial concentration and a source function are represented as complex Fourier series or a simpler combination of sinusoidal and hyperbolic functions, of which combination provides a non-zero singularity pole during the iLT.

## Supplementary information

Supplementary information.
