# Supplementary material for: Complete analytic solutions for convection-diffusion-reaction-source equations without using an inverse Laplace transform
Source: Sci Rep. 2020 May 15;10:8040. doi: 10.1038/s41598-020-63982-w (PMC7228951; doi:10.1038/s41598-020-63982-w)
Supplement: Supplementary file 1 — Supplementary information. [file 41598_2020_63982_MOESM1_ESM.pdf]

SUPPLEMENTARY INFORMATION for Complete analytic solutions for convection-diffusion-reaction-source equations without using an inverse Laplace transform

Albert S. Kim

Civil and Environmental Engineering, University of Hawaii at Manoa

Abstract

This supplementary information includes detailed derivation steps of examples, whose solutions are discussed in the main manuscript. For simplicity, analytic solutions of various sample problems were obtained altogether by deriving a full solution of the most general case.

Contents

1 Examples **2**
- 1.1 Revisit to Carslaw's example with reaction and constant boundary conditions 2
- 1.2 Revisit to Carslaw's example with reaction and zero-flux outlet 5
- 1.3 CDRS equation with a finite source function 6
  - 1.3.1 Laplace transform for steady state solution 6
  - 1.3.2 Integral factor method for steady state solution 9

2 Proofs **11**

3 Mathematical Comments on Eq. (50) **15**

1 Examples

1.1 Revisit to Carslaw's example with reaction and constant boundary conditions

We use a basic example, which was fully discussed in the literature[1, 2], by employing the initial condition

$$\mu(\xi) = \mu_0 \tag{1}$$

and boundary conditions of

$$\phi(0,\tau) = \phi_0 \tag{2}$$
$$\phi(1,\tau) = \phi_1 \tag{3}$$

which are all finite constants. A general solution after applying the constant initial condition is

$$\Phi(\xi,p) = e^{\lambda\xi}\left[B_1 e^{-\beta\xi} + B_2 e^{\beta\xi}\right] + \frac{\mu_0}{\kappa+p} \tag{4}$$

Applying boundary conditions of Eqs. (2) and (3), one calculates

$$B_1 = \frac{C_1 e^{\beta} - C_2}{2\sinh\beta} \tag{5}$$
$$B_2 = -\frac{C_1 e^{-\beta} - C_2}{2\sinh\beta} \tag{6}$$

where

$$C_1 = \frac{\phi_0}{p} - \frac{\mu_0}{\kappa+p} \tag{7}$$
$$C_2 = \left(\frac{\phi_1}{p} - \frac{\mu_0}{\kappa+p}\right) e^{-\lambda} \tag{8}$$

Substitution of Eqs. (5) and (6) into (4) yields

$$\Phi(\xi,p) = \Phi_{BC}(\xi,p) + \Phi_{IC}(\xi,p) \tag{9}$$

where Φ_{BC} and Φ_{IC} are partial solutions of Φ, originated from the boundary and initial conditions, respectively. Φ_{BC} is calculated as

$$\Phi_{BC}(\xi,p) = f(\phi_0,\phi_1,\beta,\xi)\ \frac{e^{\lambda\xi}}{p} \tag{10}$$

where

$$f(\phi_0,\phi_1,\beta,\xi) = \frac{\phi_0 \sinh(\beta(1-\xi)) + \phi_1 e^{-\lambda}\sinh(\beta\xi)}{\sinh\beta} \tag{11}$$

and, similarly, Φ_{IC} is

$$\Phi_{IC}(\xi,p) = -f(\mu_0,\mu_0,\beta,\xi)\ \frac{e^{\lambda\xi}}{\kappa+p} \tag{12}$$

where

$$f(\mu_0,\mu_0,\beta,\xi) = f(\phi_0\to\mu_0,\phi_1\to\mu_0,\beta,\xi) \tag{13}$$

Setting up $\phi_0 = 1$, $\phi_1 = 0$ and $\mu_0 = 0$ leads to the results of the previous work done by Carslaw[1, 2]. An inverse Laplace transformation of Eq. (9) provides in principle a final analytic solution of the unsteady, 1-D case:

$$\begin{aligned}\phi(\xi,\tau) = \mathcal{L}^{-1}\left[\Phi(\xi,p)\right](\xi,\tau) &= \frac{1}{2\pi i}\int_{c-i\infty}^{c+i\infty}\Phi(\xi,z)\,e^{\tau z}\mathrm{d}z \\ &= \text{Residues of } \left\{\left[e^{\tau z}\Phi_{BC}(\xi,z)\right] + \left[e^{\tau z}\Phi_{IC}(\xi,z)\right]\right\}\end{aligned} \tag{14}$$

where real p is replaced by complex variable z, and c is a real number to have the contour path integral within the region of convergence of $\Phi(\xi,\tau)$. Then, $e^{\tau z}\Phi_{BC}$ and $e^{\tau z}\Phi_{IC}$ have simple poles at $z = 0$ and $z = -\kappa$, respectively, of which residues are calculated as

$$\lim_{z\to 0}\left[z\Phi_{BC}(\xi,z)\,e^{\tau z}\right] = f(\phi_0,\phi_1,\alpha,\xi)\cdot e^{\lambda\xi}e^{0\cdot\tau} \tag{15}$$

where $\alpha = \beta(z=0) = \sqrt{\lambda^2+\kappa}$, and

$$\lim_{z\to -\kappa}\left[(z+\kappa)\,e^{\tau z}\Phi_{IC}(\xi,z)\right] = -\,f(\mu_0,\mu_0,\lambda,\xi)\ e^{\lambda\xi-\kappa\tau} \tag{16}$$

respectively. Note that both Φ_{BC} and Φ_{IC} have the same pole satisfying $\sinh\beta = 0$, equivalent to $z = -\left(\lambda^2+\kappa\right)$. The second residue of $\Phi_{BC}\left(\xi,z\right)e^{\tau z}$ from Eq. (10) has a form of

$$\lim_{z\to-\left(\lambda^2+\kappa\right)}\left[\left(z+\lambda^2+\kappa\right)\Phi_{BC}\left(\xi,z\right)e^{\tau z}\right] = -\frac{e^{\lambda\xi-\left(\lambda^2+\kappa\right)\tau}}{\lambda^2+\kappa}\,\mathcal{R}\left[f\left(\phi_0,\phi_1,\beta,\xi\right)\right] \tag{17}$$

and, similarly, that of $\Phi_{IC}\left(\xi,z\right)e^{\tau z}$ from Eq. (12) is

$$\lim_{z\to-\left(\lambda^2+\kappa\right)}\left[\left(z+\lambda^2+\kappa\right)\Phi_{IC}\left(\xi,z\right)e^{\tau z}\right] = \frac{e^{\lambda\xi-\left(\lambda^2+\kappa\right)\tau}}{\lambda^2}\,\mathcal{R}\left[f\left(\mu_0,\mu_0,\beta,\xi\right)\right] \tag{18}$$

where $\mathcal{R}\left[f\right]$ indicate the specific residue to be calculated for $z = -\lambda^2-\kappa$. Then, the final solution is obtained as

$$\phi\left(\xi,\tau\right) = f\left(\phi_0,\phi_1,\alpha,\xi\right)\cdot e^{\lambda\xi} - f\left(\mu_0,\mu_0,\lambda,\xi\right)\,e^{\lambda\xi-\kappa\tau} - \alpha^{-2}e^{\lambda\xi-\alpha^2\tau}\,\mathcal{R}\left[f\left(\phi_0,\phi_1,\beta,\xi\right)\right] + \lambda^{-2}e^{\lambda\xi-\alpha^2\tau}\,\mathcal{R}\left[f\left(\mu_0,\mu_0,\beta,\xi\right)\right] \tag{19}$$

where the residues are still unknown. One can re-apply the initial condition to have

$$\alpha^{-2}\,\mathcal{R}\left[f\left(\phi_0,\phi_1,\beta,\xi\right)\right] - \lambda^{-2}\,\mathcal{R}\left[f\left(\mu_0,\mu_0,\beta,\xi\right)\right] = f\left(\phi_0,\phi_1,\alpha,\xi\right) - f\left(\mu_0,\mu_0,\lambda,\xi\right) - \mu_0e^{-\lambda\xi} \tag{20}$$

and the final solution with all the coefficients determined for $\xi_0 < \xi < \xi_1$ is

$$\phi\left(\xi,\tau\right) = \mu_0e^{-\alpha^2\tau} + f\left(\phi_0,\phi_1,\alpha,\xi\right)\cdot\left[1-e^{-\alpha^2\tau}\right]e^{\lambda\xi} - f\left(\mu_0,\mu_0,\lambda,\xi\right)\,\left[1-e^{-\lambda^2\tau}\right]e^{\lambda\xi-\kappa\tau} \tag{21}$$

Substitution of Eqs. (11) and (13) into (21) gives

$$\begin{aligned}\phi\left(\xi,\tau\right) = {} & \frac{\phi_0\sinh\left(\alpha\left(1-\xi\right)\right)+\phi_1e^{-\lambda}\sinh\left(\alpha\xi\right)}{\sinh\alpha}\left[1-e^{-\alpha^2\tau}\right]e^{\lambda\xi} \\ & -\mu_0\left(\frac{\sinh\left(\lambda\left(1-\xi\right)\right)+e^{-\lambda}\sinh\left(\lambda\xi\right)}{\sinh\lambda}\right)\left[1-e^{-\lambda^2\tau}\right]e^{\lambda\xi-\kappa\tau}+\mu_0e^{-\alpha^2\tau}\end{aligned} \tag{22}$$

Note that the final solution $\phi\left(\xi,\tau\right)$ valid for $\xi_0 < \xi < \xi_1$ excludes the boundaries of ξ_0 and ξ_1. The steady-state solution is obtained as

$$\lim_{\tau\to\infty}\phi\left(\xi,\tau\right) = \frac{\phi_0\sinh\left(\beta\left(1-\xi\right)\right)+\phi_1\sinh\left(\beta\xi\right)}{\sinh\beta}e^{\lambda\xi}$$

which converges to Carslaw's solution[1] for $\phi_0 = 1$ and $\phi_1 = 0$:

$$\phi(\xi,\tau) = \frac{\sinh[\beta(1-\xi)]}{\sinh\beta} e^{\lambda\xi} \tag{23}$$

of which complete unsteady solution is

$$\phi(\xi,\tau) = e^{\lambda\xi} \cdot \frac{\sinh[(1-\xi)\alpha]}{\sinh\alpha} - \frac{2\pi}{\alpha} \sum_{n=1}^{\infty} \frac{n \sin(n\pi\xi)}{1+\left(\frac{n\pi}{\alpha}\right)^2} \exp\left(\lambda\xi - \tau\alpha^2 \left[1+\left(\frac{n\pi}{\alpha}\right)^2\right]\right) \tag{24}$$

if $\alpha \to \lambda$ because Eq. (24) is the analytic solution for $\kappa \neq 0$.

1.2 Revisit to Carslaw's example with reaction and zero-flux outlet

As the constant exit boundary condition of Eq. (3) is not physical, the constant-flux outlet boundary condition is considered as

$$\left[\frac{\partial\phi(\xi,\tau)}{\partial\xi}\right]_{\xi\to\xi_1} = J_1 \tag{25}$$

which includes the zero-flux condition ($J_1 = 0$) as a special case. We use a new coefficient A_i instead of B_i for $i = 1-2$ in the general solution of Eq. (4). The initial and inlet boundary conditions are identical to Eqs. (1) and (2), respectively. One can obtain

$$A_1 = \frac{C_1(\lambda+\beta)e^{\beta} - J_1 p^{-1} e^{-\lambda}}{2(\lambda\sinh\beta + \beta\cosh\beta)} \tag{26}$$

$$A_2 = \frac{J_1 p^{-1} e^{-\lambda} - C_1(\lambda-\beta)e^{-\beta}}{2(\lambda\sinh\beta + \beta\cosh\beta)} \tag{27}$$

and

$$\begin{aligned}\Phi(\xi,p) = {} & \frac{J_1 e^{-\lambda}}{p} \frac{\sinh\beta\xi}{\lambda\sinh\beta+\beta\cosh\beta} e^{\lambda\xi} + \frac{\phi_0}{p} \frac{\lambda\sinh[\beta(1-\xi)] + \beta\cosh[\beta(1-\xi)]}{\lambda\sinh\beta+\beta\cosh\beta} e^{\lambda\xi} \\ & + \frac{\mu_0}{p+\kappa} - \frac{\mu_0}{p+\kappa} \frac{\lambda\sinh[\beta(1-\xi)] + \beta\cosh[\beta(1-\xi)]}{\lambda\sinh\beta+\beta\cosh\beta} e^{\lambda\xi}\end{aligned} \tag{28}$$

The inverse Laplace transform gives the final analytic solution for the constant inlet concentration and finite exit flux:

$$\phi(\xi,\tau) = \left[J_1 e^{-\lambda} h(\alpha,\xi) + \phi_0 g(\xi)\right] e^{\lambda\xi}\left(1 - e^{-\alpha^2\tau}\right) + \mu_0 e^{-\kappa\tau}\left[1 - g(\xi) e^{\lambda\xi}\left(1 - e^{-\lambda^2\tau}\right)\right] \tag{29}$$

where

$$g(\xi) = \frac{\lambda \sinh[\alpha(1-\xi)] + \alpha\cosh[\alpha(1-\xi)]}{\lambda\sinh\alpha + \alpha\cosh\alpha} \tag{30}$$

$$h(\xi) = \frac{\sinh\alpha\xi}{\lambda\sinh\alpha + \alpha\cosh\alpha} \tag{31}$$

For the conventional zero-flux of $J_1 = 0$, one obtains

$$\phi(\xi,\tau) = \phi_0 g(\xi) e^{\lambda\xi}\left(1 - e^{-\alpha^2\tau}\right) + \mu_0 e^{-\kappa\tau}\left[1 - g(\xi) e^{\lambda\xi}\left(1 - e^{-\lambda^2\tau}\right)\right] \tag{32}$$

which satisfies $\phi(\xi, 0) = \mu_0$.

1.3 CDRS equation with a finite source function

1.3.1 Laplace transform for steady state solution

Governing equation for the convection, diffusion, reaction and source is

$$\frac{\partial^2\Phi}{\partial\xi^2} + \frac{\partial\Phi}{\partial\xi} + (1-p)\Phi = -\frac{\sigma}{p} \tag{33}$$

which is simplified to

$$-\frac{\sigma}{p} = \left[(D+\lambda_1)(D+\lambda_2)\right]\Phi \tag{34}$$

where $\lambda_1 = \frac{1}{2}(1 - i\gamma)$, $\lambda_2 = \frac{1}{2}(1 + i\gamma)$, $\left(\frac{\gamma}{2}\right)^2 = \frac{3}{4} - p$, and $\gamma = 2\sqrt{\frac{3}{4} - p}$ for Zhong et al.'s case[3].

One can define

$$\Phi_2 = (D + \lambda_2)\Phi \tag{35}$$

to have

$$-\frac{\sigma}{p} = (D+\lambda_1)\,\Phi_2 = e^{-\lambda_1\xi}\frac{\partial}{\partial\xi}\left(e^{\lambda_1\xi}\Phi_2\right) \tag{36}$$

and

$$\Phi_2 = -\frac{1}{p}e^{-\lambda_1\xi}\int^{\xi}\sigma(\eta)\,e^{\lambda_1\eta}\mathrm{d}\eta \tag{37}$$

Using the mathematical identity:

$$\sigma = e^{-a\xi}\sin(b\xi) = \frac{e^{-a\xi}}{2i}\left(e^{ib\xi}-e^{-ib\xi}\right) \tag{38}$$

where $a=\pi/2$ and $b=5\pi$, one can further calculate Φ_2 as

$$\Phi_2 = -\frac{1}{2ip}\left[\frac{e^{-(a-ib)\xi}}{(\lambda_1-a+ib)} - \frac{e^{-(a+ib)\xi}}{(\lambda_1-a-ib)}\right] \tag{39}$$

Using the representation of Φ_2, one can solve for Φ using

$$\Phi_2 = (D+\lambda_2)\,\Phi = e^{-\lambda_2\xi}\frac{\partial}{\partial\xi}\left(e^{\lambda_2\xi}\Phi\right) \tag{40}$$

by following the procedure below and

$$\Phi = \frac{1}{2ip}\left[\frac{e^{-(a-ib)\xi}}{\left(a-ib-\frac{1}{2}(1+i\gamma)\right)\left(\frac{1}{2}(1-i\gamma)-a+ib\right)} - \frac{e^{-(a+ib)\xi}}{\left(a+ib-\frac{1}{2}(1+i\gamma)\right)\left(\frac{1}{2}(1-i\gamma)-a-ib\right)}\right] \tag{41}$$

and therefore

$$2ip\Phi = -\frac{e^{-(a-ib)\xi}}{\left(\tilde{a}-i\left(b+\frac{1}{2}\gamma\right)\right)\left(\tilde{a}-i\left(b-\frac{1}{2}\gamma\right)\right)} + \frac{e^{-(a+ib)\xi}}{\left(\tilde{a}+i\left(b-\frac{1}{2}\gamma\right)\right)\left(\tilde{a}+i\left(b+\frac{1}{2}\gamma\right)\right)} \tag{42}$$

where $\tilde{a}=a-\frac{1}{2}$. In short,

$$-2ip\Phi e^{a\xi} = \frac{\left(\tilde{a}+i\left(b+\frac{1}{2}\gamma\right)\right)\left(\tilde{a}+i\left(b-\frac{1}{2}\gamma\right)\right)}{\left(\tilde{a}^2+\left(b+\frac{1}{2}\gamma\right)^2\right)\left(\tilde{a}^2+\left(b-\frac{1}{2}\gamma\right)^2\right)}e^{ib\xi} - \frac{\left(\tilde{a}-i\left(b+\frac{1}{2}\gamma\right)\right)\left(\tilde{a}-i\left(b-\frac{1}{2}\gamma\right)\right)}{\left(\tilde{a}^2+\left(b+\frac{1}{2}\gamma\right)^2\right)\left(\tilde{a}^2+\left(b-\frac{1}{2}\gamma\right)^2\right)}e^{-ib\xi} \tag{43}$$

and therefore

$$-2ip\Phi e^{a\xi}\left(\tilde{a}^2+\left(b+\tfrac{1}{2}\gamma\right)^2\right)\left(\tilde{a}^2+\left(b-\tfrac{1}{2}\gamma\right)^2\right)=\left[\tilde{a}^2+\left(\frac{\gamma}{2}\right)^2-b^2\right]2i\sin b\xi+i2b\tilde{a}\cdot 2\cos b\xi \tag{44}$$

Then, Φ is finally simplified to

$$\Phi=e^{-a\xi}\frac{\left[b^2-\tilde{a}^2-\left(\frac{\gamma}{2}\right)^2\right]\sin b\xi-2b\tilde{a}\cdot\cos b\xi}{p\left(\tilde{a}^2+\left(b+\frac{1}{2}\gamma\right)^2\right)\left(\tilde{a}^2+\left(b-\frac{1}{2}\gamma\right)^2\right)} \tag{45}$$

again the coefficients are $a=\frac{\pi}{2}$,$\tilde{a}=a-\frac{1}{2}=\frac{\pi-1}{2}$, $\gamma=2\sqrt{\frac{3}{4}-p}$, $\gamma_0=\sqrt{3}$, and $b=5\pi$. The particular solution at the steady state is obtained as

$$\phi_{P,ss}=e^{-a\xi}\frac{\left[b^2-\tilde{a}^2-\left(\frac{\gamma_0}{2}\right)^2\right]\sin b\xi-2b\tilde{a}\cdot\cos b\xi}{\left(\tilde{a}^2+\left(b+\frac{1}{2}\gamma_0\right)^2\right)\left(\tilde{a}^2+\left(b-\frac{1}{2}\gamma_0\right)^2\right)} \tag{46}$$

and simplifying the denominator gives

$$\phi_{P,ss}=e^{-a\xi}\frac{\left[b^2-\tilde{a}^2-\left(\frac{\gamma_0}{2}\right)^2\right]\sin b\xi-2b\tilde{a}\cdot\cos b\xi}{\left(b^2-\tilde{a}^2-\left(\frac{\gamma_0}{2}\right)^2\right)^2+(2b\tilde{a})^2} \tag{47}$$

and further

$$\phi_{P,ss}=e^{-a\xi}\frac{[\sin b\xi\ \cos\theta_0-\cos b\xi\ \sin\theta_0]}{|Z_0|}=e^{-a\xi}\frac{\sin(b\xi-\theta_0)}{|Z_0|} \tag{48}$$

where

$$|Z_0|=\sqrt{\left(b^2-\tilde{a}^2-\left(\frac{\gamma_0}{2}\right)^2\right)^2+(2b\tilde{a})^2} \tag{49}$$

and

$$\theta_0 = \tan\left(\frac{2\tilde{a}b}{b^2 - \tilde{a}^2 - \left(\frac{\gamma_0}{2}\right)^2}\right) \tag{50}$$

The transient exponent is

$$z = \frac{3}{4} + \tilde{a}^2 - b^2 + i\left(2b\tilde{a}\right) \tag{51}$$

which is valid for both

$$\tilde{a}^2 + \left(b \pm \frac{1}{2}\gamma\right)^2 = 0 \tag{52}$$

in other words

$$b^2 - \tilde{a}^2 - \left(\frac{\gamma}{2}\right)^2 = i\left(2b\tilde{a}\right) \tag{53}$$

1.3.2 Integral factor method for steady state solution

In this section, we use the conventional integral factor method to independently derive the steady-state concentration of Zhong et al.'s case[3] discussed above. The dimensionless governing equation in real space is

$$\frac{\partial^2\phi}{\partial\xi^2} + \frac{\partial\phi}{\partial\xi} + \phi = -e^{-a\xi}\sin\left(b\xi\right) \tag{54}$$

where $a = \frac{\pi}{2}$ and $b = 5\pi$ are given in section 1.3.1. Eq. (54) is rewritten as

$$\left(\mathcal{D}_\xi + \lambda_m\right)\left(\mathcal{D}_\xi + \lambda_p\right)\phi = e^{-a\xi}\sin\left(b\xi\right) \tag{55}$$

where $\lambda_p = \left(1 + i\sqrt{3}\right)/2$ and $\lambda_m = \left(1 - i\sqrt{3}\right)/2$. Now, one can define

$$\psi \equiv \left(\mathcal{D}_\xi + \lambda_p\right)\phi \tag{56}$$

and substitute ψ into Eq. (55) to derive

$$\psi = e^{-\lambda_m\xi}C_m + \frac{1}{2i}\left[\frac{e^{-(a-ib)\xi}}{\lambda_m - a + ib} - \frac{e^{-(a-+ib)\xi}}{\lambda_m - a - ib}\right] \tag{57}$$

where C_m is an integral constant to be determined. Substitution of Eq. (57) in to (55) provides

$$\phi(\xi) = b_1 e^{-\lambda_p \xi} + b_2 e^{-\lambda_m \xi} + \frac{e^{-a\xi}}{2i}\left[\frac{e^{ib\xi}}{Z_1} - \frac{e^{-ib\xi}}{Z_1^*}\right] \tag{58}$$

where B_1 and B_2 are unknown constants and

$$Z_1 = (\lambda_m - a + ib)(\lambda_p - a + ib) \tag{59}$$

and Z_1^* is the complex conjugate of Z_1. One can rewrite Z_1 as

$$Z_1 = \left(\tilde{a}^2 + \tfrac{3}{4} - b^2\right) - 2i\tilde{a}b \tag{60}$$

$$= |Z_1| e^{i\theta_1} \tag{61}$$

where

$$|Z_1|^2 = \left(\tilde{a}^2 + \tfrac{3}{4} - b^2\right)^2 + (2\tilde{a}b)^2 \tag{62}$$

$$\theta_1 = \tan^{-1}\left(\frac{2\tilde{a}b}{b^2 - \tilde{a}^2 - \frac{3}{4}}\right) \tag{63}$$

and $\tilde{a} = a - \frac{1}{2}$. It is worth noting that Z_1 of Eq. (62) and Z_0 of Eq. (49) are equal to each other and so does θ_0 of Eq. (50) and θ_1 of Eq. (63). For simplicity, Z_0 and θ_0 are used throughout the manuscript including this supplementary information. Then, the steady-state solution ϕ of Eq. (58) is rewritten as

$$\phi(\xi) = e^{-\xi/2}\left[b_1 \sin\left(\tfrac{\sqrt{3}}{2}\xi\right) + b_2 \cos\left(\tfrac{\sqrt{3}}{2}\xi\right)\right] + \frac{e^{-a\xi}\sin(b\xi - \theta_0)}{|Z_0|} \tag{64}$$

Note that the last term in the right-hand-side of Eq. (64) is identical to $\phi_{P,ss}$ of Eq. (48). By applying the boundary conditions of the zero concentration, i.e., $\phi(\xi = 0) = 0$ and $\phi(\xi = 1) = 0$, one drives the coefficient B_1 and B_2 of Eq. (64):

$$b_1 = \frac{\sin\theta_0}{|Z_0|} \tag{65}$$

$$b_2 = -\frac{\sin\theta_0 \, \cos\left(\frac{\sqrt{3}}{2}\right) + e^{-\tilde{a}}\sin(b - \theta_0)}{|Z_0|} \tag{66}$$

2 Proofs

Proof of Eq. (34)

$$\begin{aligned}
-\frac{\sigma}{p} &= \left(D^2 + D + (1-p)\right)\Phi \\
&= \left(D^2 + D + \frac{1}{4} + \left(\frac{3}{4} - p\right)\right)\Phi \\
&= \left(\left(D + \frac{1}{2}\right)^2 + \left(\frac{\gamma}{2}\right)^2\right)\Phi \\
&= \left[\left(D + \frac{1}{2}(1 - i\gamma)\right)\left(D + \frac{1}{2}(1 + i\gamma)\right)\right]\Phi \\
&= \left[(D + \lambda_1)(D + \lambda_2)\right]\Phi
\end{aligned}$$

Proof of Eq. (39)

$$\begin{aligned}
\Phi_2 &= -\frac{1}{p}e^{-\lambda_1 \xi}\int^{\xi}\frac{e^{-a\eta}}{2i}\left(e^{ib\eta} - e^{-ib\eta}\right)e^{\lambda_1 \eta}\mathrm{d}\eta \\
&= -\frac{1}{2ip}e^{-\lambda_1 \xi}\int^{\xi}\left(e^{ib\eta} - e^{-ib\eta}\right)e^{(\lambda_1 - a)\eta}\mathrm{d}\eta \\
&= -\frac{1}{2ip}e^{-\lambda_1 \xi}\int^{\xi}\left(e^{(\lambda_1 - a + ib)\eta} - e^{(\lambda_1 - a - ib)\eta}\right)\mathrm{d}\eta \\
&= -\frac{1}{2ip}e^{-\lambda_1 \xi}\left[\frac{e^{(\lambda_1 - a + ib)\xi}}{(\lambda_1 - a + ib)} - \frac{e^{(\lambda_1 - a - ib)\xi}}{(\lambda_1 - a - ib)}\right] \\
&= -\frac{1}{2ip}\left[\frac{e^{-(a - ib)\xi}}{(\lambda_1 - a + ib)} - \frac{e^{-(a + ib)\xi}}{(\lambda_1 - a - ib)}\right]
\end{aligned}$$

Proof of Eq. (41)

$$\begin{aligned}
\Phi &= e^{-\lambda_2 \xi} \int^{\xi} \Phi_2(\eta)\, e^{\lambda_2 \eta} \mathrm{d}\eta \\
&= -\frac{1}{2ip} e^{-\lambda_2 \xi} \int^{\xi} \left[\frac{e^{-(a-ib-\lambda_2)\eta}}{(\lambda_1 - a + ib)} - \frac{e^{-(a+ib-\lambda_2)\eta}}{(\lambda_1 - a - ib)} \right] \mathrm{d}\eta \\
&= -\frac{1}{2ip} e^{-\lambda_2 \xi} \left[\frac{e^{-(a-ib-\lambda_2)\xi}}{-(a-ib-\lambda_2)(\lambda_1 - a + ib)} - \frac{e^{-(a+ib-\lambda_2)\xi}}{-(a+ib-\lambda_2)(\lambda_1 - a - ib)} \right] \\
&= \frac{1}{2ip} e^{-\lambda_2 \xi} \left[\frac{e^{-(a-ib-\lambda_2)\xi}}{(a-ib-\lambda_2)(\lambda_1 - a + ib)} - \frac{e^{-(a+ib-\lambda_2)\xi}}{(a+ib-\lambda_2)(\lambda_1 - a - ib)} \right] \\
&= \frac{1}{2ip} \left[\frac{e^{-(a-ib)\xi}}{\left(a - ib - \frac{1}{2}(1+i\gamma)\right)\left(\frac{1}{2}(1-i\gamma) - a + ib\right)} - \frac{e^{-(a+ib)\xi}}{\left(a + ib - \frac{1}{2}(1+i\gamma)\right)\left(\frac{1}{2}(1-i\gamma) - a - ib\right)} \right]
\end{aligned}$$

Proof of Eq. (42)

$$\begin{aligned}
2ip\Phi &= \frac{e^{-(a-ib)\xi}}{\left(a - ib - \frac{1}{2}(1+i\gamma)\right)\left(\frac{1}{2}(1-i\gamma) - a + ib\right)} - \frac{e^{-(a+ib)\xi}}{\left(a + ib - \frac{1}{2}(1+i\gamma)\right)\left(\frac{1}{2}(1-i\gamma) - a - ib\right)} \\
&= \frac{e^{-(a-ib)\xi}}{\left(a - \frac{1}{2} - ib - i\frac{1}{2}\gamma\right)\left(\frac{1}{2} - a - i\frac{1}{2}\gamma + ib\right)} - \frac{e^{-(a+ib)\xi}}{\left(a - \frac{1}{2} + ib - \frac{1}{2}i\gamma\right)\left(\frac{1}{2} - a - i\frac{1}{2}\gamma - ib\right)} \\
&= \frac{e^{-(a-ib)\xi}}{\left(a - \frac{1}{2} - i\left(b + \frac{1}{2}\gamma\right)\right)\left(\frac{1}{2} - a + i\left(b - \frac{1}{2}\gamma\right)\right)} - \frac{e^{-(a+ib)\xi}}{\left(a - \frac{1}{2} + i\left(b - \frac{1}{2}\gamma\right)\right)\left(\frac{1}{2} - a - i\left(b + \frac{1}{2}\gamma\right)\right)} \\
&= -\frac{e^{-(a-ib)\xi}}{\left(a - \frac{1}{2} - i\left(b + \frac{1}{2}\gamma\right)\right)\left(-\frac{1}{2} + a - i\left(b - \frac{1}{2}\gamma\right)\right)} + \frac{e^{-(a+ib)\xi}}{\left(a - \frac{1}{2} + i\left(b - \frac{1}{2}\gamma\right)\right)\left(-\frac{1}{2} + a + i\left(b + \frac{1}{2}\gamma\right)\right)} \\
&= -\frac{e^{-(a-ib)\xi}}{\left(\left(a - \frac{1}{2}\right) - i\left(b + \frac{1}{2}\gamma\right)\right)\left(\left(a - \frac{1}{2}\right) - i\left(b - \frac{1}{2}\gamma\right)\right)} + \frac{e^{-(a+ib)\xi}}{\left(\left(a - \frac{1}{2}\right) + i\left(b - \frac{1}{2}\gamma\right)\right)\left(\left(a - \frac{1}{2}\right) + i\left(b + \frac{1}{2}\gamma\right)\right)} \\
&= -\frac{e^{-(a-ib)\xi}}{\left(\tilde{a} - i\left(b + \frac{1}{2}\gamma\right)\right)\left(\tilde{a} - i\left(b - \frac{1}{2}\gamma\right)\right)} + \frac{e^{-(a+ib)\xi}}{\left(\tilde{a} + i\left(b - \frac{1}{2}\gamma\right)\right)\left(\tilde{a} + i\left(b + \frac{1}{2}\gamma\right)\right)} \qquad (67)
\end{aligned}$$

Proof of Eq. (44)

$$
\begin{aligned}
& -2ip\Phi e^{a\xi}\left(\tilde{a}^2+\left(b+\tfrac{1}{2}\gamma\right)^2\right)\left(\tilde{a}^2+\left(b-\tfrac{1}{2}\gamma\right)^2\right) \\
&=\left(\tilde{a}+i\left(b+\tfrac{1}{2}\gamma\right)\right)\left(\tilde{a}+i\left(b-\tfrac{1}{2}\gamma\right)\right)e^{ib\xi}-\left(\tilde{a}-i\left(b+\tfrac{1}{2}\gamma\right)\right)\left(\tilde{a}-i\left(b-\tfrac{1}{2}\gamma\right)\right)e^{-ib\xi} \\
&=\left[\tilde{a}^2-\left(b+\tfrac{1}{2}\gamma\right)\left(b-\tfrac{1}{2}\gamma\right)+i\tilde{a}\left(b-\tfrac{1}{2}\gamma+b+\tfrac{1}{2}\gamma\right)\right]e^{ib\xi}-\left[\tilde{a}^2-\left(b+\tfrac{1}{2}\gamma\right)\left(b-\tfrac{1}{2}\gamma\right)-i\tilde{a}\left(b-\tfrac{1}{2}\gamma+b+\tfrac{1}{2}\gamma\right)\right]e^{-ib\xi} \\
&=\left[\tilde{a}^2-\left(b+\tfrac{1}{2}\gamma\right)\left(b-\tfrac{1}{2}\gamma\right)+i2b\tilde{a}\right]e^{ib\xi}-\left[\tilde{a}^2-\left(b+\tfrac{1}{2}\gamma\right)\left(b-\tfrac{1}{2}\gamma\right)-i2b\tilde{a}\right]e^{-ib\xi} \\
&=\left[\tilde{a}^2+\left(\frac{\gamma}{2}\right)^2-b^2+i2b\tilde{a}\right]e^{ib\xi}-\left[\tilde{a}^2+\left(\frac{\gamma}{2}\right)^2-b^2-i2b\tilde{a}\right]e^{-ib\xi} \\
&=\left[\tilde{a}^2+\left(\frac{\gamma}{2}\right)^2-b^2\right]2i\sin b\xi+i2b\tilde{a}\cdot 2\cos b\xi
\end{aligned}
$$

Proof of Eq. (51)

$$
\begin{aligned}
\tilde{a}^2+\left(b\pm\frac{1}{2}\gamma\right)^2 &= 0 \\
\pm\gamma &= 2\left(-b+i\tilde{a}\right) \\
\pm 2\sqrt{\frac{3}{4}-p} &= 2\left(-b+i\tilde{a}\right) \\
\frac{3}{4}-p &= \left(-b+i\tilde{a}\right)^2 \\
p &= \frac{3}{4}-\left(-b+i\tilde{a}\right)^2 \\
&= \frac{3}{4}-\left(b^2-i2b\tilde{a}-\tilde{a}^2\right) \\
&= \frac{3}{4}-\left(b^2-\tilde{a}^2\right)+i2b\tilde{a} \\
z &= \left(\frac{3}{4}+\tilde{a}^2-b^2\right)+i\left(2b\tilde{a}\right)
\end{aligned}
$$

Proof of Eq. (53)

$$\begin{aligned}
b^2 - \tilde{a}^2 - \left(\frac{\gamma}{2}\right)^2 &= b^2 - \tilde{a}^2 - \left(\sqrt{\frac{3}{4} - p}\right)^2 \\
&= b^2 - \tilde{a}^2 - \left(\sqrt{-\tilde{a}^2 + b^2 - i\left(2b\tilde{a}\right)}\right)^2 \\
&= b^2 - \tilde{a}^2 - \left(-\tilde{a}^2 + b^2 - i\left(2b\tilde{a}\right)\right) \\
&= b^2 - \tilde{a}^2 + \tilde{a}^2 - b^2 + i\left(2b\tilde{a}\right) \\
&= i\left(2b\tilde{a}\right)
\end{aligned} \tag{68}$$

3 Mathematical Comments on Eq. (50)

Eq. (50) can be re-written as

$$\phi(\xi,\tau) = \frac{1}{2\pi i}\int_{c-i\infty}^{c+i\infty} f(\xi,z)\,\mathrm{d}z = \frac{1}{2\pi i}\int_{c-i\infty}^{c+i\infty} \frac{g(\xi,z)}{z}\mathrm{d}z \tag{69}$$

where

$$f(\xi,z) = z^{-1}\,g(\xi,z) = e^{\tau z}\Phi(\xi,z) \tag{70}$$

$$g(\xi,z) = e^{\tau z}\Lambda(\xi,z) \tag{71}$$

$$\Lambda(\xi,z) = z\,\Phi(\xi,z) \tag{72}$$

Here, we consider that $g(\xi,z)$ is analytic on and within the integral range that includes $z = z_0\,(=0)$, and $g(\xi,z_0)$ is non-zero, finite. We apply the residue theorem to obtain

$$g(\xi,z_0) = \frac{1}{2\pi i}\int_{c-i\infty}^{c+i\infty} \frac{g(\xi,z)}{z-z_0}\mathrm{d}z = e^{\tau z_0}\Lambda(\xi,z_0) = \phi_{\mathrm{ss}}(\xi) \tag{73}$$

which indicates that $g(\xi,z_0)$ is mathematically the residue of $f(\xi,z)$ at $z = z_0$ (not that of $g(\xi,\tau)$), and physically the steady-state solution in the limit of $\tau \to \infty$. If the full solution $\phi(\xi,\tau)$ consists of steady-state and transient terms, then Eq. (50) can be alternatively represented as

$$\phi(\xi,\tau) = \frac{1}{2\pi i}\int_{c-i\infty}^{c+i\infty} e^{\tau z}\left[\frac{g_0(\xi,z)}{z-z_0} + \frac{g_1(\xi,z)}{z-z_1}\right]\mathrm{d}z \tag{74}$$

$$= g_0(\xi,z_0) + e^{\tau z_1}g_1(\xi,z_1) \tag{75}$$

where z_1 is negative in most engineering and scientific applications. It is worth noting that the identities of $g(\xi,z_0) = g_0(\xi,z_0) = \phi_{\mathrm{ss}}(\xi)$, but the inequality of $g(\xi,z) \neq g_0(\xi,z)$. In addition, applying boundary condition results in $g_1(\xi,z_1) = \mu(\xi) - g_0(\xi,z_0)$, and finally gives in a complex form:

$$\phi(\xi,\tau) = g_0(\xi,z_0) + e^{\tau z_1}\left[\mu(\xi) - g_0(\xi,z_0)\right] \tag{76}$$

which is equivalent to Eq. (55). Note that $z_0 = 0$ and $z_1 \neq 0$ are the singularity poles of $f(\xi,z)$, and therefore, g, g_0, and g_1 are considered as equivalent to coefficients of Laurent's series of $f(\xi,z)$.

References

[1] Mohsen, Mohammad Farrukh N. and Baluch, Mohammed H., "An Analytical Solution of the Diffusion--Convection Equation Over a Finite Domain", *Applied Mathematical Modelling* 7, 4 (1983), pp. 285--287. https://doi.org/10.1016/0307-904x(83)90084-7.

[2] Carslaw, H. S. & Jaeger, J. C. *Conduction of Heat in Solids (Oxford Science Publications)* (Oxford University Press, 1986). https://www.xarg.org/ref/a/0198533683/.

[3] Zhong, J., Zeng, C., Yuan, Y., Zhang, Y. & Zhang, Y. Numerical Solution of the Unsteady Diffusion-Convection-Reaction Equation Based on Improved Spectral Galerkin Method. *AIP Advances* 8, 045314 (2018). https://doi.org/10.1063/1.5023332.
